# Human Perceptions of Megafaunal Extinction Events Revealed by Linguistic Analysis of Indigenous Oral Traditions

**DOI:** 10.1007/s10745-018-0004-0

**Published:** 2018-06-04

**Authors:** Priscilla M. Wehi, Murray P. Cox, Tom Roa, Hēmi Whaanga

**Affiliations:** 1 Centre for Sustainability, University of Otago, PO Box 56, Dunedin, 9054 New Zealand; 2 Te Pūnaha Matatini, Manaaki Whenua – Landcare Research, 764 Cumberland St, Private Bag 1930, Dunedin, 9054 New Zealand; 3 Te Pūnaha Matatini, Statistics and Bioinformatics Group, Institute of Fundamental Sciences, Massey University, Private Bag 11 222, Palmerston North, 4442 New Zealand; 4 Faculty of Māori and Indigenous Studies, University of Waikato, Private Bag 3105, Hamilton, 3240 New Zealand

**Keywords:** Cultural evolution, Indigenous resource management, Megafauna, Moa, Oceania, New Zealand, Maori, Socio-ecological systems, Traditional ecological knowledge

## Abstract

Human settlement into new regions is typically accompanied by waves of animal extinctions, yet we have limited understanding of how human communities perceived and responded to such ecological crises. The first megafaunal extinctions in New Zealand began just 700 years ago, in contrast to the deep time of continental extinctions. Consequently, indigenous Māori oral tradition includes ancestral sayings that explicitly refer to extinct species. Our linguistic analysis of these sayings shows a strong bias towards critical food species such as moa, and emphasizes that Māori closely observed the fauna and environment. Temporal changes in form and content demonstrate that Māori recognized the loss of important animal resources, and that this loss reverberated culturally centuries later. The data provide evidence that extinction of keystone fauna was important for shaping ecological and social thought in Māori society, and suggest a similar role in other early societies that lived through megafaunal extinction events.

**Electronic supplementary material:**

The online version of this article (10.1007/s10745-018-0004-0) contains supplementary material, which is available to authorized users.

## Introduction

Island ecosystems act as natural laboratories for understanding the processes of extinction (Carlquist 1974; Prebble and Wilmshurst 2009), including the role of, and impact upon, human communities. The Pacific Ocean was one of the last regions settled by humans, and its extinction timeline is therefore more recent than on the continents. However, even in the Pacific, most major extinction phases occurred well before written records. Consequently, although we know a great deal about the science of extinction events from archaeological and other data, we still understand little about how human communities perceived, and responded to, the resulting ecological crises, or the development of community conservation ‘rules’ and actions by these communities. By applying a combination of quantitative and qualitative linguistic methods, we have explored indigenous responses to major faunal extinctions in one commonly used form of oral tradition, *whakataukī*. In New Zealand, settled as recently as AD 1280 (Wilmshurst *et al.* 2008; Perry *et al.* 2014), the strong oral tradition among indigenous Māori includes a series of ancestral sayings (*whakataukī*) that provide glimpses into the island’s early extinction events and their importance as cultural signposts. In generalized form, our findings may shed light on how other societies became aware of, and responded to, earlier animal extinctions across Eurasia and the Americas.

In common with other Pacific island environments, the arrival of humans in New Zealand resulted in high rates of extinction among its predominantly avian fauna (Duncan *et al.* 2002; Bromham *et al.* 2012). The species most affected were those especially vulnerable to human hunters, including the group of large, flightless birds known as moa [Aves: Dinornithiformes] (Holdaway 1989; Worthy 1997; Cassey 2001). Detailed reconstruction of Holocene bird fauna indicates that approximately 28 land bird species became extinct on the two main islands of New Zealand in the 500 years between initial settlement by Māori (c. AD 1280) and first European contact (AD 1769) (Worthy and Holdaway 2002; Tennyson 2006; Wood 2013). Berkes (2008) argues that ecological crises trigger learning in human communities, which in turn shapes subsequent resource management practices. However, few studies have explored the development of conservation learning in response to ecological crises, and despite the rapid loss of major avian megafauna in New Zealand less than 700 years ago (Holdaway and Jacomb 2000; Tennyson 2006; Allentoft *et al.* 2014; Perry *et al.* 2014) how this ecological change affected cultural learning remains essentially unknown.

Quantitative analysis of linguistic markers to determine the timing and evolution of manuscripts has been increasingly employed over the last 20 years (Barbrook *et al.* 1998; Spencer *et al.* 2004; Eagleton and Spencer 2006; Howe and Windram 2011). More recently, these analyses have been extended to investigate the cultural legacies of folk tale records, some of which likely originated before the emergence of written records (da Silva and Tehrani 2016). The contexts in which communications take place also shape linguistic form and length (Wray and Grace 2007). Linguistic theory suggests that communication within close family groupings is dominated by implicit meaning. This contrasts with the cues that dominate exoteric language used by distantly related groups, such as increased length and transparency (Wray and Grace 2007). Ecological information conveyed in ancestral sayings during early human settlement phases in a new land is thus likely embedded implicitly in short phrases*.* Here, we use linguistic, historical and structural cues in a body of *whakataukī* to analyze the development of socioecological thought over a period of c.650 years from the time of Polynesian arrival in New Zealand.

We first hypothesised that large bodied animals, such as the flightless moa, would predominate in Māori *whakataukī* if food sources were an important preoccupation for these settlers. Avian body size is a significant predictor of hunting intensity across the Pacific (Duncan *et al.* 2002), and moa – a group of ratites that ranged from the size of a turkey to much larger than an ostrich – were a primary food and tool resource for the Polynesian ancestors of the indigenous Māori people of New Zealand (Anderson 1989), given the lack of native mammals in New Zealand. Second, we predicted that the form and content of *whakataukī* over time would reflect rapid and ongoing socioenvironmental evolution associated with new settlement (Wray and Grace 2007). We expected that *whakataukī* length would reflect social change. Specifically, we hypothesized that early settlement in close-knit family groupings, where relevance and implied contexts were well-understood within the group would result in shorter *whakataukī*; whereas in the changing environments of rapid settlement expansion and the formation of larger sub-tribal and tribal groupings, alliances, and warfare the context of *whakataukī* would be less well understood and they would, on average, increase in length (Wray and Grace 2007). Finally, we predicted that major negative environmental change, such as the loss of critical megafaunal food species, should drive a progression from immediate observations of loss to a generalized understanding of the causes of extinction and finally to the deployment of explicit ecological management practices in keeping with the development of conservation practice (Best 1904; Berkes 2008; Bowman *et al.* 2015).

## Materials and Methods

Prior to European arrival in the late seventeenth century, Māori was a pre-literate language with a strongly developed oral tradition and a large unwritten literature of songs, poetry, and proverbs as in many indigenous cultures. This tradition uses *whakataukī*, as well as narratives (*pūrākau*) that contain philosophical thought, metaphor (*kupu whakarite*), epistemological constructs, cultural codes, worldviews, and song (*waiata*) in everyday life. *Whakataukī* formed an important part of this tradition (Mead and Grove 2001; Wehi 2009). It is important to note that although the translation of *whakataukī* as ‘ancestral sayings’ suggests an association with historic oral tradition, these sayings are still widely used by orators and speakers of Māori today.

### Dataset

European settlement in NZ was initiated shortly after 1800, and gathered momentum in the second half of the nineteenth century (Fig. 2). During this period, many early European ethnographers recorded and compiled Māori oral tradition, including Grey, Colenso, Smith, White, Williams, Best, and Firth (Supplementary Materials). These source materials, along with other archived records, were comprehensively compiled, revised, translated, and interpreted by Mead and Grove (2001) with the later addition of translations and interpretations. We used this pariemological dataset of 2669 *whakataukī* as our primary dataset, supplemented by similar entries from other compilations (total *n* = 3421; see electronic supplementary materials for details). From this dataset, we analysed 657 *whakataukī* that explicitly refer to fauna.

### Time Attribution

Drawing from Mead’s (1984) classification of the temporal development of Māori artistry, and Davidson’s (1984) cultural phases, each faunal *whakataukī* was assigned to one of five broad time periods: before AD 1350 (pre-dating Māori settlement of New Zealand), 1350-1500 (the very early New Zealand settlement period), 1500-1650 (rapid settlement expansion), 1650-1800 (intertribal fighting and early European contact) and post-1800 (the period following European contact). These time periods equate approximately, in English literature terms, to the early Anglo-Saxon period of Beowulf (~1000 AD); to that of Chaucer (~1343-1400); Shakespeare (1564-1616); Jonathan Swift (1667-1745); and Charles Dickens (1812-1870). To assign faunal *whakataukī* to these temporal categories, we called upon TR, a Māori linguist, translator, historian, and native speaker, to derive estimated chronological dates for the *whakataukī* without any prior knowledge of the hypotheses of the study (see below for validation) so that we could establish relationships both between different versions of the same *whakataukī*, and between *whakataukī* with different content (Table 1).

**Table 1:** Examples showing how linguistic cues, historical and cultural context, and identification of events and ancestor names inform chronological dating of *whakataukī*

Example	Theme	English translation	Time period	Explanation
(a) Moa				
1. Mate ā-moa	Extinction	Dead as the moa	1500-1650	The first seven examples have similar content or core meanings, but display shifts in language structure (to a greater or lesser extent). For instance, example (1) is the most compact and the only example to use the stative verb mate (be dead) with the ‘ā-prefix/noun’ combination to derive a stative adjective; we believe this is the oldest of these eight *whakataukī*. Examples (2) and (3) are similar to example (1), using the ‘stative/ā-prefix/noun’ combination, but additional information is included: the stative verb *ngaro* (be missing, lost, hidden, extinct), the particle *ka* to mark tense/aspect and a nominal phrase to indicate subject – the difference in subject being *te iwi nei* ‘this tribe’ and *te tangata* ‘the person/the Māori people’. Example (3) and the following example express the widely held belief in the late nineteenth century that Māori would similarly become extinct. Examples (4) and (5) are structurally different from the first three examples, but very similar to each other. They include the stative verb *ngaro* and the prepositional phrase *i te ngaro o te moa*, but are marked with different tense/aspect particles – *ka* vs. *kua* ‘perfect tense’. The perfect tense marker in this case implies the achievement of a state as the result of an event. On the other hand ‘Ka’ indicates the past. Thus, example (5) was used for people suddenly killed or carried off by death. Example (6), however, is not marked by any tense/aspect marker, it introduces the stative verb *huna* (be concealed, unnoticed) and includes the prepositional phrase *i te huna a te moa*. Mead suggests the saying expresses contempt at the poor concealment ability of moa. However, it also refers to the disappearance of a social group, or aspect of culture. This could alternatively suggest an element of disbelief that something so ubiquitous could disappear. Example (7) is structurally different from the rest: it uses the stative *huna* in an emphatic construction with the particle *ko* with the prepositional phrase *i te moa*. Example (8) describes an ecological observation in which the *moa*, a giant herbivore browser with up to 5 kg of gizzard stones that were used to break down fibrous plant material, were compared with gluttonous people, who similarly browse great quantities of food. Example (9) describes the rātā, a parasitic forest tree, that fails to stand upright independently, and is susceptible to trampling while young. For example (10), the boughs, leaves and flowers of the koromiko tree may have been used to cover the moa flesh when cooked in an umu (a ground oven).
2. Ka ngaro ā-moa te iwi nei	Extinction	This tribe will disappear like the moa	1800-	
3. Ka ngaro ā-moa te tangata	Extinction	The Māori will become extinct like the moa	1800-	
4. Ka ngaro i te ngaro o te moa	Extinction	Lost like the loss of the moa	1800-	
5. Kua ngaro i te ngaro o te moa	Extinction	Perished as the moa perished	1500-1650	
6. Huna i te huna a te moa	Extinction	Hidden as the moa hid	1800-	
7. Ko te huna i te moa!	Extinction	It is like the disappearance of the moa	1650-1800	
8. He puku moa!	Ecological observation	A stomach of a moa!	1350-1500	
9. He rātā te rākau i takahia e te moa	Ecological observation	A rātā was the tree trampled by the moa	1500-1650	
10. He koromiko te wahie i taona ai te moa	Food preparation	Koromiko is the wood with which the moa was cooked	1500-1650	
(b) Fish				
Hā! He ika poto te ika nei!	Historical	What! A short fish, this one’	1500-1650	Specific historical details form the context for this saying, and can thus be used to help identify its chronology. Awakanoi of Ngāti Awa was slain by near Rūātoki. According to Best (1925), when the body was turned over to reveal its identity, the victor uttered this saying. It apparently meant he had hoped for a more prominent victim. Another explanation is that Ipuhue was disappointed that the victim had not provided more of a contest.
(c) Chicken				
Ai pī	Breeding	Chicken breeding.	before 1350	Referring to a prolific parent with numerous children, this *whakataukī* has been identified as pre-dating Māori settlement. Overpopulation is a recurring rationale for Māori departure from Hawaiki in oral tradition. Domestic chickens either were not carried, or did not survive on canoes during the journey to Aotearoa New Zealand.

TR used linguistic and structural cues, vocabulary identifications, historical contexts and embedded references to ancestor names, events, and genealogies to make these temporal assignments (Roa 2016) (Table 1). For example, specific historical details form the context for the *whakataukī* on fish shown in Table 1. In other *whakataukī*, estimated dates of species arrivals (e.g., for pītongatonga, after European arrival) and old and modern word usages and transliterations (e.g., pī and heihei) were used to cross reference dating.

### Validation of Time Period Assignments

Scoping tests demonstrate that expert linguistic training is necessary for accurate dating over and above native-speaker and community elder status (Chipere 2000). As a result, we devised a blind validation test to confirm the ability of our language expert and co-author (TR) that used Māori sentences with no temporal context taken from two time periods (late nineteenth century, early twenty-first century) to obtain a reliability value for distinguishing early and late sentence structures dating from approximately a century apart. We presented TR with a randomized set of sentences of comparable length (approximately 15 – 50 words) from oral recordings in the Māori language, with 100 examples selected from each of two time periods (Table S4).

Sentences were selected from six speakers of Māori who were recorded in the 1940s, but were born around the 1870s. These are regarded as the landmark early Māori oral recordings and have been used extensively in other comparative studies (Keegan *et al.* 2014). These were compared with sentences taken from 58 Māori speakers who were recorded on Te Karere (a news and current affairs television program broadcast on state television in Māori) that aired in late November/December 2015. The program’s content focuses on topics of national significance to the targeted Māori audience.

The mobile unit of the National Broadcasting Service made recordings of Māori speakers between 1946 and 1948. The Unit made three tours: two in the North Island, covering the West Coast from Wanganui to Waitara in late 1946, and the Waikato and Thames Valley districts in 1947, and one in the South Island to the Otago region in 1948. Sentences were selected in the following way. First, for the speakers born in the 1870s, we demarcated each new topic of conversation, and selected the first sentence after the third sentence that was between 15 and 50 words in length. Each sentence was checked for any obvious words or dates that would provide the informant with explicit temporal information (e.g., references to World War II). If the first example was deemed unsuitable, or if parts were inaudible, the next example of 15-50 words was selected and checked for suitability. For the second set of speakers, we excluded sentences spoken by the interviewer as these are normally scripted rather than representing impromptu speech. We then selected the first sentence of the interviewee of between 15 and 50 words in length. A maximum of five sentences was selected from a single speaker (Table S4).

Language expert TR achieved a hit rate of 81% and error rate of 19%, yielding a sensitivity index *d’* of 1.76. Sentences from the early period were assigned with slightly more accuracy (86%) than sentences from the late period (76%). The null hypothesis that the language expert assigns sentences randomly to the early and late periods can therefore be rejected both for the set of early sentences (${\upchi}_1^2$ = 13.4, *P* = 0.00025) and the set of late sentences (${\upchi}_1^2$ = 28.2, *P* = 0.00000011). Given this discriminatory power for two time periods separated by only 100 years, the *whakataukī* were likely assigned to five much wider time periods with similar or better accuracy.

### Knowledge Development

We classified *whakataukī* according to Berkes’ (2008) mechanisms of knowledge development (i.e., observation and monitoring, trial and error experimentation, learning from other places and times, and knowledge encoded in language and other narratives) to examine key developments in traditional knowledge, practice, and resource management over these time periods. In addition, we examined *whakataukī* using a model proposed by Crombie (1985a, b, 1987) and Whaanga (2006) that identifies mechanisms of human thinking and language structure based on cognitive processes. The model uses four main distinctions (temporal, additive, associative, and causal) to explore connections between the development of knowledge through time and language structure shifts and time periods. To make these classifications, the model identifies discourse relations, and the presence and absence of relational signaling and encoding (i.e., coherence, cohesive devices, co-ordination, subordination, conjuncts, and lexis) with linguistic and world knowledge (i.e., prior understandings) (Roa 2016; Supplementary Materials). Using these classification techniques, we were able to establish relationships between different versions of the same *whakataukī*, and *whakataukī* with different content.

### Word Frequencies

Qualitative analyses included inspection of word frequencies in *whakataukī*. All function words (such as ‘a,’ ‘the,’ and ‘in’) were removed from the dataset using standard UNIX operations (particularly the command line program ‘grep’). Word frequencies were determined using an online word counting tool (http://www.textfixer.com/tools/online-word-counter.php; accessed April 2017).

### Bird Data

We used Dunning (2007) to determine mean weights for all bird species (Table S1, Supplementary Materials), and then regressed these weights against total word occurrence for that species or group in the *whakataukī* faunal dataset. Prevalence of bird species across New Zealand archaeological sites (Fig. 2) was obtained from Worthy (1999)*.*

### Use of Māori and English Bird Names

**Fig. 1:**
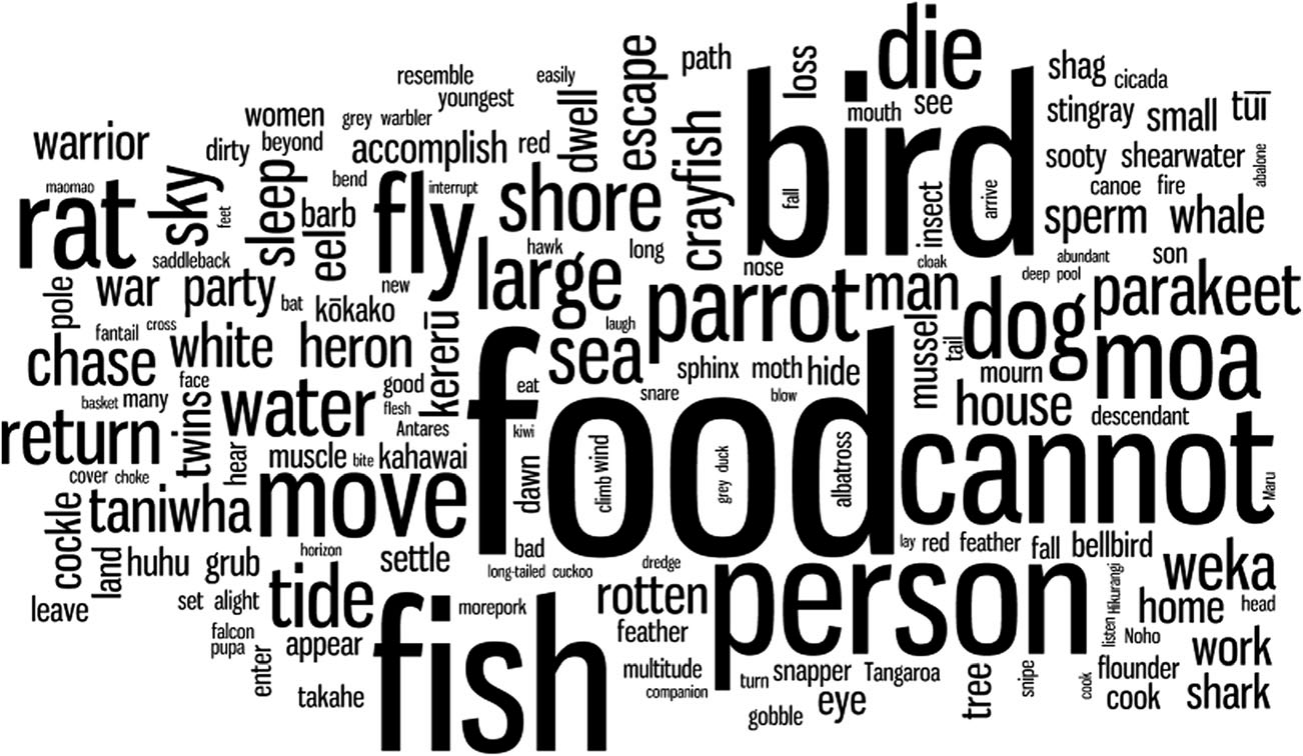
Relative frequency of words in the faunal subset of Māori *whakataukī*, translated here into English. Function words have been removed

We have used bird and plant names that are common usage in New Zealand to refer to species reported in this paper. In some cases these are Māori (e.g., kiwi, miro), and in some cases English (e.g., blue duck). We have not italicized these names, but provide the appropriate scientific name on first usage to assist the reader with identification. In addition, all scientific names for birds represented in Fig. 1 along with their average weights, and all scientific names for birds that became extinct on the two main islands of New Zealand, have been provided in Tables S1 and S2.

**Fig. 2:**
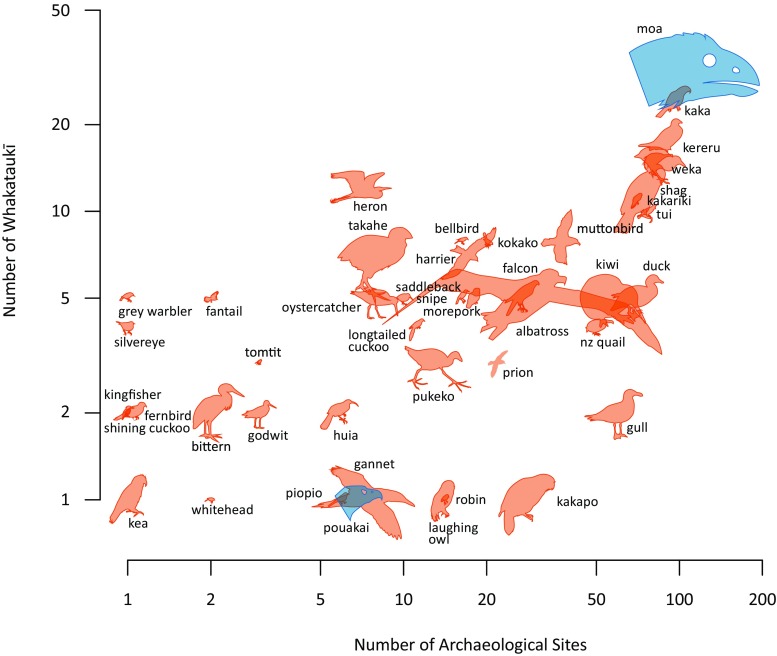
Large birds (pictures scaled according to size; Table S1) are discussed more often than small birds in *whakataukī* and are found at a larger number of archaeological sites. Moa are heavily represented in *whakataukī*; a moa head only is shown due to their disproportionately large size. Birds represented in blue (i.e.moa and pouakai) became extinct prior to European arrival – but other extinct birds do not occur in the *whakataukī* and are thus not shown in the figure (Table S2). Data from archaeological sites are from Worthy (1999), shown with permission

We note that Māori names do not always map cleanly to modern taxonomic units. Bird names in Fig. 2 may therefore refer to closely related and morphologically similar species, such as moa, albatross, shag, kiwi, and kākāriki (native parakeets). Morphologically similar birds from the same genus that are now described as different species on the North and South Islands are often described by a single common name, such as ‘snipe,’ ‘saddleback,’ and ‘kōkako.’ In Fig. 2, some bird names have also been shortened for visual clarity: ‘bittern’ refers to the New Zealand bittern, ‘godwit’ to the bar-tailed godwit, ‘oystercatcher’ to the variable oystercatcher, ‘heron’ to the white heron, and ‘quail’ to the extinct New Zealand quail. Macrons are not shown for names on the plot.

### Historical Reports of Moa

We considered whether the *whakataukī* referring to moa and their extinction that were identified as dating to the nineteenth century were in part a product of the intense scientific interest that arose near that time about this bird. Anderson (1989) has inferred that the controversy generated within the scientific community about the discovery of moa, and its apex predator the Haast’s Eagle, likely preceded or stimulated the recording of sayings about moa during the post European period, and suggests that the moa were as common a symbol in public imagery then as the kiwi is today. However, a closer evaluation of the evidence suggests otherwise (Supplementary Materials). Governor George Grey asserted that Māori all knew the word moa as ‘a bird well known to their ancestors.’ Grey also recorded the lament of Ikaherengutu that includes references to moa, and was sung by Te Wherowhero on the death of his brother (see Supplementary Materials for further details). Because Anderson’s survey of oral tradition was primarily limited to narratives (*pūrākau*) a number of critical references to moa in *waiata* (song) and *whakataukī* were not identified.

### Statistical Analyses

All statistical approaches were implemented in R (R Development Core Team 2010).

## Results

Among the faunal set of *whakataukī*, the most frequent word is ‘food’ (*kai*), indicating that subsistence was indeed a high priority (Fig. 1). As predicted, large birds are more frequently mentioned in *whakataukī* than small birds (*n* = 657 *whakataukī*; *r* = 0.60, *P* < 0.0001, Fig. 2), consistent with the same trend whereby large birds are more commonly found in archaeological sites than small birds (*n* = 112 sites; *r* = 0.41, *P* = 0.0052; Fig. 2).

Few *whakataukī* were dated to pre-1350 or post-1800. Nonetheless, *whakataukī* from the early settlement period are shorter (mean length 6.75 words) than *whakataukī* from the subsequent phase of rapid settlement expansion (AD 1500-1650, mean length 9.13 words; t_304_ = −5.10, *P* < 0.001). No increase in word length was seen when we compared period 4 (1650-1800, prior to European arrival) and period 5 (1800-, after European arrival) (mean lengths 9.56 and 10.4 words for periods 4 and 5 respectively, t_58_ = −0.859, *P* = 0.39). In addition, qualitative examination of early *whakataukī* show they are dominated by implicit meanings indicative of closely related family groupings in contrast to *whakataukī* from later periods in which larger tribal groupings, alliances, and warfare prevail. For example, the brief phrase ‘ai pī’ references overpopulation as a recurring rationale for Polynesian voyaging through the Pacific (Table 1).

**Fig. 3:**
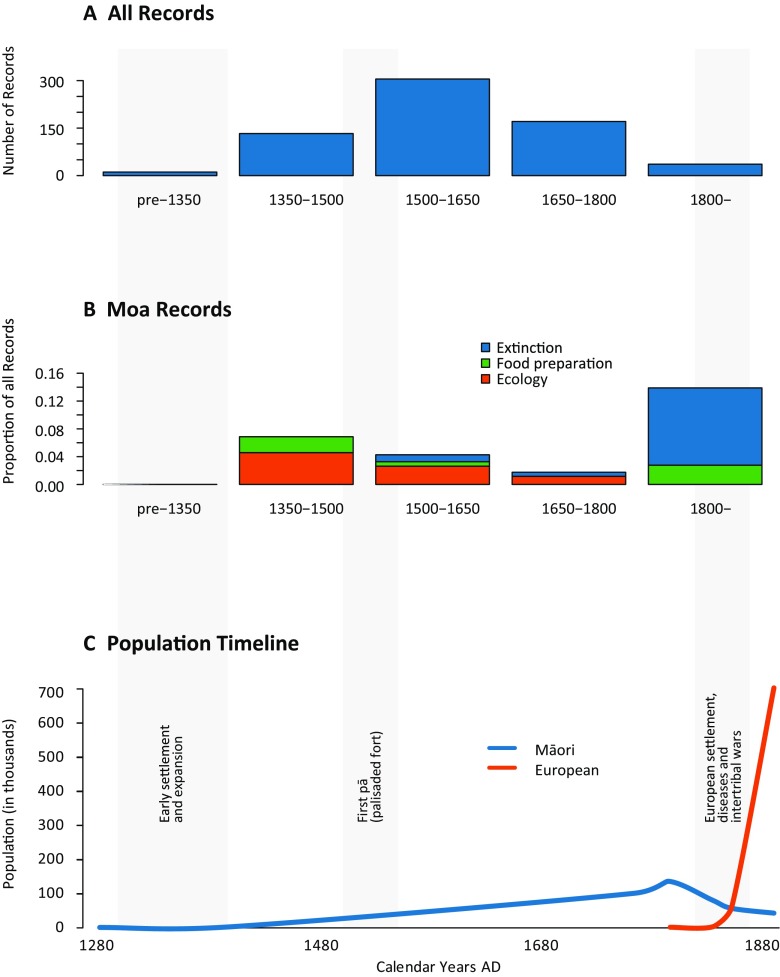
*Whakataukī* records in relation to time. **a** *Whakataukī* that refer to all fauna occur most frequently between AD 1500-1650. **b** The abundance and likely importance of moa *whakataukī* varies through time, as shown by their relative proportion to all faunal *whakataukī* during each time period. **c** The Polynesian founder population c. AD 1280 is generally estimated as <400 in size (Whyte *et al.* 2005), and population expansion was slow in comparison to rapid European settlement in the nineteenth century (Holdaway *et al.* 2014)

**Fig. 4:**
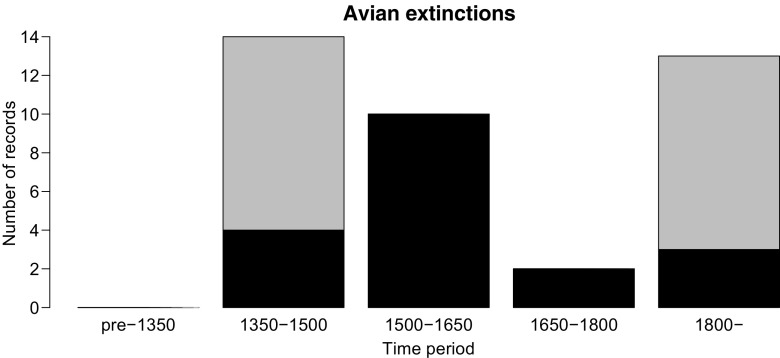
Most avian extinctions on mainland New Zealand occurred either prior to AD 1500 (within 150 years of Māori arrival), or after European arrival post-1800 AD. ‘Moa’ is treated here as a name for all nine species of moa, reflecting its indigenous usage; if this group of nine species is treated as one taxonomic unit, the loss of names would be commensurately higher for species in the 1350-1500 time period. See Table S2 for estimated extinction dates. Key avian extinction periods occurred shortly after Māori and European settlement periods. Grey represents names birds for which the Māori name is no longer known, and black represents bird species or groups for which the Māori name has been retained

Moa form an archetypal group that are strongly over-represented in *whakataukī* as in the archaeological record (Figs. 2 and 3). *Whakataukī* about moa comprise 4.6% of all faunal *whakataukī*, and 9.8% of all *whakataukī* that specifically mention birds. In striking contrast, other large extinct megafauna, such as the endemic geese and adzebills with adult weights >15 kg, are not represented in the *whakataukī*. Indeed, their original Māori names are largely lost (Fig. 4). Māori names for species that became extinct prior to European arrival are now unknown, with the exception of words for moa and pouākai. This loss contrasts with the retention of names for avifauna that became extinct after European arrival. Only one other extinct bird with remarkable body size is mentioned – the Haast’s Eagle (*Aquila moorei*), a giant raptor with a 3 m wingspan called *pouakai* or *hokioi* by Māori. This eagle is the only known apex predator of moa other than humans (Anderson 1989) and quickly followed the moa into extinction.

**Fig. 5:**
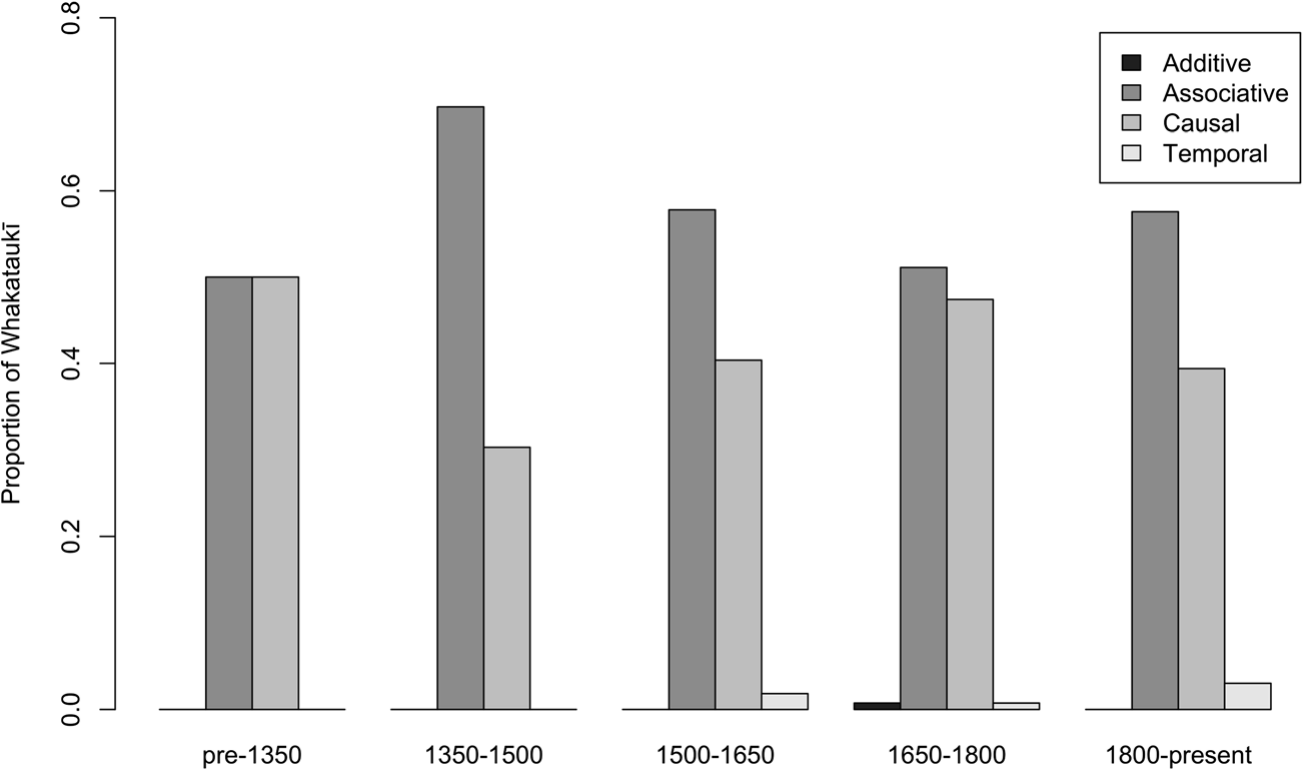
*Whakataukī* shift from a predominance of observations early on, towards a rise in causal sayings from 1500 to 1800, before a further small rise in associative observations after 1800

Qualitative analysis of the dataset indicates that the nature and frequency of moa *whakataukī* are disproportionally skewed toward two time periods (1350-1500, and post-1800) (Fig. 3). Three main themes emerge from the moa *whakataukī*: ecological information, food preparation, and concerns about extinction (Fig. 3, Table 1). Ecological observations are especially evident during the early settlement period. The two peaks of *whakataukī* about moa reflect i) moa ecology and their likely functional extinction around AD 1400-1450, and ii) archetypal links to ideas of extinction for Māori themselves (Table 1) during the social upheaval that followed European colonisation in the early to mid-nineteenth century. Almost all moa *whakataukī* post-1800 link moa disappearance to impending Māori extinction. In general, the *whakataukī* dataset demonstrates a trend from simple observation in the early settlement period to awareness of causal agency in later periods (AD 1500-1800; Fig. 5), as expected for the development of traditional knowledge, resource management practices, and conservation ‘rules’ (Berkes 2008).

## Discussion

The strong relationship between bird size, frequency in the oral record, and frequency in recorded material from archaeological sites indicates that despite the limitations of each of these datasets Māori both targeted and talked about animal species (in this case, birds) that were important food resources. Species that hold strong cultural significance, such as the white heron (*Ardea modesta)*, are also well represented in the *whakataukī*. While it is difficult to fully test our hypothesis because, for example, small bones are less likely to be found in archaeological deposits, the relationship shown here is clear for all larger bird species.

The frequency of references to moa in the *whakataukī* provides a powerful contrast to that of other large bird species that became extinct before European arrival. Some large species, such as adzebills, may have had restricted ranges, and therefore might be expected to appear less. Nonetheless, the general absence of references to extinct birds in *whakataukī* emphasises the language loss that frequently accompanies biodiversity extinction (Maffi 2005).

Given the likely speed with which moa became extinct (<150 years) (Perry *et al.* 2014) it is unsurprising that *whakataukī* on moa food preparation and on extinction appear to be contemporaneous in the oral record. Diverse and emotive language emphasises the ‘loss’ and ‘death’ of moa, suggesting that the extinction of moa was widely noted and discussed. A later set of moa *whakataukī* appears after European arrival in the nineteenth century and almost uniformly employs the loss of moa as a metaphor for the feared extinction of Māori. This re-mapping of *whakataukī* concerning the fifteenth century loss of moa to a much later nineteenth century social crisis – the imminent and very real threat of Māori biological and cultural extinction – powerfully emphasizes the impact of moa on the cultural psyche of Māori. The frequency and content of these later *whakataukī* support the view that Māori were not only aware of the dismal end met by moa, but also that moa extinction came to serve as an archetypal exemplar for extinction more generally.

*Whakataukī* word lengths in the early period support the predictions of esoteric and exoteric language theory (Wray and Grace 2007), which expected linguistic forms and structures to change with communication context. Early *whakataukī* indeed carry strongly embedded meanings that reflect close family groupings in contrast to *whakataukī* from later periods, with larger tribal groupings, which have greater transparency of meaning. That is, interactions with strangers, language contact, and stratification of society all influence language evolution and the way that ecological material is presented. These findings align with the results of the dating methodology, providing confidence in the temporal assignment of language cues and structures identified for each period.

Tracking changes in resource management practices proved challenging using *whakataukī*. Qualitative analysis reveals a nuanced understanding of ecological relationships in faunal *whakataukī* from later time periods, for example noting seasonal linkages between species (Table S3, Supplementary Materials). This is consistent with the finding that terms associated with complex social relationships and structures, such as chieftainship and territoriality, increase in frequency through time (Wehi *et al.* 2013). Nevertheless, the progression from ecological observations to adaptive management is not clearly reflected in this dataset. One possibility is that the nuances of adaptive management practices are too complex for the brief format of *whakataukī* (median 8 words, range 2-36 words, Tables 1 and S3). Notably, specific management practices are not explicitly discussed in any of the faunal *whakataukī* even though environmental stewardship was widely noted during the nineteenth century by early European writers (Best 1904; Kawharu 2000; Kitson and Moller 2008). For instance, harvests of sooty shearwater (*tītī*) have operated continuously for several centuries (Hawke *et al.* 2003) with defined sustainability practices passed down inter-generationally (Kitson and Moller 2008) and the management of harvests falling largely under the responsibility of chiefs (Best 1904; Kawharu 2000). Our focus on faunal *whakataukī* may have excluded *whakataukī* that mention ecosystem-wide management. Ecological management events and environmental frameworks may also be more conspicuous in other forms of oral culture, such as storytelling (Bowman *et al.* 2015). Alternatively, observation may not necessarily have led to immediate or visible action, perhaps as reflected in the unexpected absence of most large extinct birds in the *whakataukī*.

Oral tradition, such as these *whakataukī* passed down by Māori, provide our only real glimpses into the ecological relationships and concerns of early settler populations, and provide early human context to an otherwise relatively dry scientific record of extinction events. The *whakataukī* emphasise that indigenous peoples are not simply passive actors against an environmental backdrop but rather interact with the environment in myriad ways that affect not only the species assemblages present but also the development of cultural values, ideas, and practices. As such, these *whakataukī* provide evidence of the links between cultural and biological diversity (Maffi 2005). Similar linguistic analysis of other indigenous oral traditions globally could illuminate the development of socioecological world views and conservation learning in other cultures, at least where extinction events are relatively recent. They provide our closest available proxy to the thoughts and responses of the human communities that lived through the big megafaunal extinctions, which occurred very early in human history on the continents.

## Electronic supplementary material

ESM 1 (DOCX 104 kb)
