# Supplementary material for: Human Perceptions of Megafaunal Extinction Events Revealed by Linguistic Analysis of Indigenous Oral Traditions
Source: Hum Ecol Interdiscip J. 2018 Jun 4;46(4):461–70. doi: 10.1007/s10745-018-0004-0 (PMC6133014; doi:10.1007/s10745-018-0004-0)
Supplement: Supplementary file 1 — (DOCX 104 kb) [file 10745_2018_4_MOESM1_ESM.docx]

Supplementary Materials for

Human perceptions of megafaunal extinctions revealed by linguistic analysis of indigenous oral traditions

P.M. Wehi*, M.P. Cox, T. Roa, and H. Whaanga

*Corresponding author. E-mail: wehip@landcareresearch.co.nz

Materials and Methods

Tables S1 to S4

References

Materials and Methods

**Knowledge development:**

We classified the *whakataukī* according to a four-way distinction based on cognitive processes proposed by Crombie (1985a, b, 1987) and Whaanga (2006) (shown in Figure 5) to explore connections between the development of knowledge through time, and language structure shifts and time periods (Berkes 2008). This distinction is based on the relationships that link two or more meaning propositions together, where ‘a proposition … is an abstraction made up of a semantic predicator (an action, state or process) and one or more arguments that relate to it’ (Whaanga 2007). The four-way distinction of temporal, causal, associative and additive was derived from tracking the inventories proposed by a number of linguists (Whaanga 2006). The temporal category links relationships in time (i.e., *Temporal Sequence* and *Temporal*

*Overlap*). The causal category links causal, conditional and concession relationships (i.e., causal: *Reason-Result*, *Grounds-Conclusion*, *Means-Result*, *Means-Purpose*; conditional: *Realisable Condition*, *Unrealisable Condition*; concession: *Concession-Contraexpectation*). The associative category links comparison, contrast and alternation (choice) relationships (i.e., comparison: *Paraphrase, Statement-Affirmation, Simple Comparison, Exemplification*; contrast: *Simple Contrast,* *Statement-Denial, Denial-Correction, Exception, General-Particular*; alternation: *Supplementary Alternation, Contrastive Alternation*). The additive relation does not involve time, cause and effect or the matching of propositions in terms of comparison, contrast or choice, but links additive relationships. The encoding and signaling of these relationships can occur in a range of grammar and lexis including co-ordination, subordination, conjuncts, nouns, etc. For example, in Māori the reason member of a *Reason-Result* relation may be signaled by a subordinating conjunction (e.g., *nā te mea* ‘because’), a noun (e.g., *take* ‘reason’), a complex preposition (e.g., *i runga* ‘because’) or a causative prefix (e.g., *whaka*-)*.* To make these classifications, the model identifies the encoding and signaling of these relationships, considers coherence (how the parts of text are logically and systematically organized in an orderly fashion so that the reader / listener can understand the entire text), and cohesion (the ways texts are tied together by linguistic devices), with linguistic and world knowledge (that is, prior understandings, pragmatic knowledge and general knowledge about culture and daily life) (Roa 2016).

**Historical reports of moa:**

Early reports of moa, and deposits of moa bones, intrigued early European explorers and settlers such as Colenso, Polack, Dieffenbach, Taylor and Mantell. It is probable the first moa bone to reach an osteological authority was collected in the 1830s (Anderson 1989); the trader Joel Polack, for example, was shown a number of fossilized moa bones by Māori in 1838 near Mt Hikurangi in the North Island (Polack 1838). Richard Owen identified a new species of giant, flightless bird on the basis of an unpromising 15 cm length of femur sent to him by John Rule (Owen 1839). Moa were, however, not formally reported to the scientific community until 1843. Although Owen’s deduction was initially ridiculed, later discoveries of bone showed him to be correct, and a scientific frenzy about this giant, extinct group of birds ensued. Speculation about the existence of moa was rife in some sections of New Zealand and European society from the 1840s, for example in *The New Zealand Journal* (London) in 1844 (Anderson 1989). Interest in moa bone discoveries reached their height in the late 19^th^ century, with notable finds in 1864 (Owen 1879), 1870 (Hutton and Coughtrey 1875), 1874 (Buller 1888), 1878 (Owen 1879), 1884, and 1894 (Anderson 1989). Sir Julius Haast made his first public pronouncement on the subject of the moa’s extinction in his presidential address to the Philosophical Institute of Canterbury in 1871, and a great deal of debate around ‘the moahunters’ took place in the 1870s and subsequently.

Initial evidence could be interpreted to agree with Anderson’s hypothesis. Anderson identified the first use of the term ‘moa’ as from 1838, in stories told by East Coast Māori to Williams and Colenso, and was unable to find any earlier usage of the term. Furthermore, he concurred with Best (Best 1942) that Māori stories did not provide reliable evidence of moa ecology or biology, on the basis of the evidence before him. Colenso believed that moa had apparently escaped the attention of Māori, and that ‘fabulous stories’ were the only evidence of moa in oral tradition (Colenso 1844). Anderson’s survey of oral tradition, however, was primarily limited to narratives (pūrākau); a number of critical references to moa in waiata (song) and whakataukī (ancestral sayings) were therefore not identified. In addition, Buick (1937) noted that Colenso lived in a region where moa were probably less prevalent than in other parts of the country, and was therefore limited in the generality of his suppositions.

There is also a strong body of contrasting evidence. This evidence includes that of Grey (1870), who collected some of the whakataukī included in our reference dataset, and who asserted that twenty five years previously, Māori all knew the word moa, as ‘a bird well known to their ancestors’. He records, for example, the lament of Ikaherengutu (of Ngāti Ruanui, born circa 1760s) for the loss of his children, some of whom had died in battle and others through natural causes. This lament was sung by Te Wherowhero (the first Māori King) on the death of his brother Te Kati. The verse referencing moa reads:

*Kei te wareware, taku ngākau,*

*Ngā hanga a te rau.*

*Mei kaiā rānei, te marama*

*I mate ai?*

*Mei kaiā rānei, te pari*

*I horo ai?*

*Ngā huri nei i pirau ai?*

*Mei taua mea, ka ruru ngā atua*

*Ki a tātou,*

*Ka ngaro i te ngaro a te moa.*

Let not my heart forget

The exploits of many.

Was the Moon plundered (like me)

When it died?

Was the cliff-side plundered (like me)

When it fell?

Or the seed kūmara that they have rotted?

If such be it, verily, the gods have united

Against us all,

And lost are we, lost like the moa

Since Grove and Mead completed their magnificent collection of Māori ancestral sayings, with translations, and notes on meaning (Grove 1981, Mead and Grove 1989, 1991, 1994 and 2001), we have been able to evaluate this aspect of oral tradition more comprehensively. The dating of Māori ancestral sayings in this paper challenges the view that Māori did not know of moa, or use this term prior to the 19^th^ century.

The expertise of one of us (TR) as an exponent and repository of oral tradition, respected throughout NZ, also challenges this view. We therefore present an alternate view to Anderson’s suggestion that names for moa such as te manu a Ruakapanga, manu-whakatau and kuranui are not ‘convincing’ (Anderson 1989). We emphasize again that our comprehensive analysis of embedded meaning in ancestral moa sayings will go some way to updating views on the ecological relevance of Māori oral tradition (see Wehi 2009 for examples from a plant species), as could also occur with other indigenous traditions.

**Biographies of major 19^th^ century ethnographers**:

**George Grey** (1812-1898) is regarded as one of the earliest recorders of Māori oral tradition having arrived in New Zealand in 1845 to serve as Governor (he served two terms from 1845 to 1853 and again from 1861 to 1868). Between 1845 and 1854, Grey commissioned Wiremu Maihi Te Rangikāheke (who is of Te Arawa, Ngāti Rangiwewehi and Ngāti Kererū descent), and other Māori writers such as Piri Kawau, Matene Te Whiwhi and Tamihana Te Rauparaha, to produce manuscripts documenting oral tradition including autobiographical material, Māori knowledge and language, genealogy, prayers, historic events, customs, traditional arts, leadership and social structure focusing on the events of the late 17^th^ and 18^th^ centuries (Biggs 1952; Curnow 1985; Grey 1971; Simmons 1966). The works of Grey and Te Rangikāheke, along with those commissioned pieces and other works collected by Grey, primarily from the tribal areas of Te Arawa, Ngāti Kahungunu, Waikato, Ngāti Toa, Ngāti Raukawa, and Te Atiawa, exceeds 9,800 pages of manuscript, of which only 196 pages of prose and 500 pages of poetry have been printed (Simmons 1966). Te Rangikāheke’s produced a large volume of work - 21 sole author manuscripts (670 pages), contributions to 17 collaborative manuscripts (100 pages), and ten letters reporting of political matters spanning successive governors (68 pages). Curnow (1985) notes that “there is no evidence that he ever wrote or spoke English”, and although Te Rangikāheke was a major contributor to Grey’s works on songs (Grey 1853) and proverbs (Grey 1857), his contribution was not acknowledged by the author.

**John White** (1826-1891), scholar, writer, linguist, public servant, emigrated to New Zealand from Cockfield in Durham England in 1834. His family settled at Mata in Hokianga where his family established a farm and trading enterprise. Towards the end of the 1830s, White and his younger brother were sent to England to finish their schooling, returning to New Zealand in October 1840. At Hokianga, White spent much of his time among local Māori experts who taught him the language, Māori lore and customs. He began ‘collecting local Ngā Puhi material, which would one day form the basis of his ethnographic fictions. This material included ‘New Zealand songs’ (waiata and karakia, some of which White translated into English), ‘native tales’ (tara or stories told to him by Ngā Puhi friends, which White recorded in Māori and sometimes translated), ‘New Zealand anecdotes’ (short descriptions or observations in English illustrating Ngā Puhi life and custom), and longer narratives, often involving accounts of warfare, drawn from Ngā Puhi oral history’ (O’Leary 2008). In the late 1840s, White gained the attention of the governor, George Grey, by sending him manuscripts of Māori traditions, and when White moved to Auckland in 1851, he became Grey’s secretary and translator (Foster 2011). Between 1875 and 1878 White edited *Te Wananga,* a Māori newspaper published by Henare Tomoana (of Ngāti Kahungunu and Ngāti Te Whatu-i-apiti descent) (Reilly 2014). Reilly notes that ‘[t]his experience and the reputation White had acquired as an ethnographer resulted in his appointment in early 1879 as the compiler and writer of an official Maori history. The task took over 10 years, from 10 April 1879 to 30 September 1890’ (Reilly 2014). This work was published in six volumes between 1887 and 1890 as *The ancient history of the Maori, his mythology and traditions* (White 1887-1890).

**William Colenso** (1811-1899), printer, botanist, explorer and politician, travelled to New Zealand from Cornwell England to work for the Church Missionary Society as a printer/missionary. Colenso established a printer in Paihia, Bay of Islands New Zealand in the 1830s and by 1837 he printed New Zealand’s first book, the Māori New Testament (Bagnall et al. 2012). By 1840, the printer had produced more than 74,000 copies of various pamphlets and books, not all religious publications, including the Treaty of Waitangi. Colenso undertook numerous major explorations to expand the missionary vision into isolated Māori communities and to collect botanical species and Māori oral tradition. Extending on his journeys within Northland, he travelled extensively through the North Island including trips through the East Coast, Urewera, and Waikato districts in 1841–1842. During this journey he collected nearly 1,000 specimens and wrote an account of his journey and botanical discoveries in *Memoranda of an Excursion, made in the Northern Island of New Zealand in the summer of 1841–42* (Colenso 1844). Later in 1844, he traversed the coastal districts of the East Coast from Hicks Bay to Tūranga (Gisborne) where he took a ship bound for Wellington. Due to bad weather the ship was forced to land at Castle Point where he turned inland to visit the Urewera district where he conducted a census of the remote villages. From there he followed the Whakatāne River valley to the Bay of Plenty and returned home via Tauranga, the Waikato, and Otahuhu. That same year he was ordained a deacon and appointed to open a new mission station at Ahuriri in Hawkes Bay, which extended from Taupo to Wellington and eastward to the Ruahine and Tararua Ranges (Byrnes 2014). He crossed the ranges on five occasions describing some of his experiences in *An Account of visits to the Ruahine Mountain Range* (Colenso 1884). During his life, Colenso founded the printing industry in New Zealand, recorded many Māori oral traditions (although he was often undiplomatic and insensitive to their traditions) (e.g. Colenso 1868, 1878, 1879), and made a significant contribution to science in New Zealand, publishing numerous papers on a wide range of biological and ethnological topics in the *Transactions of the New Zealand Institute* (Byrnes 2014). He was elected a Fellow of the Linnaean Society (FLS) in 1865, and in 1886 was elected as a Fellow of the Royal Society (FRS).

**Percy Smith** (1840-1922), a surveyor, public servant, ethnologist and writer, emigrated to New Zealand from Suffolk, England with his family when he was 9 years old. He attended school at New Plymouth and then Omata, leaving in 1854 to help on the family farm. In 1855, Smith joined the provincial survey department as a cadet assisting in the subdivision of land around the settlement of New Plymouth (Byrnes 2014). Smith worked as a professional surveyor throughout the northern half of the North Island surveying areas such as Kaipara and Northern Wairoa districts, Thames, Waikato, Taranaki, Auckland, Hawke’s Bay, Taupō, Rotorua and Pitt Island in the Chatham Islands (Cowan 1935). He later became chief surveyor and commissioner of Crown lands in the Auckland district, and from 1889 until his retirement in 1900, he was surveyor general and secretary for lands and mines. During his many surveying expeditions, Smith travelled to many remote areas collecting and recording Māori oral traditions, culture and histories (Best 1923; Byrnes 2014; Tregear 1922). Tregear noted that it was whilst surveying the bush lands surrounding New Plymouth in the 1850s that Smith ‘began to acquire a knowledge of the Māori language, and his efforts to obtain a mastery over that language were so persistent that he came to be regarded as one of the most accomplished Māori scholars in the Dominion’. Smith was co-founder with Tregear of the Polynesian Society in 1892, and was co-editor of the *Journal of the Polynesian Society* until his death in 1922. He was a recognized scholar of the Māori language, and was considered by his contemporaries to be a leading Pākehā authority on the history and traditions of the Māori, publishing a large number of articles, books, monographs and pamphlets on Polynesian history, mythology, customs and tribal lore (Best 1923). His major works in this area include *Hawaiki: The original home of the Maori* (Smith 1898, 1904a), *Wars of the northern against the southern tribes of New Zealand in the nineteenth century* (Smith 1904b), *History and traditions of the Maoris of the West Coast, North Island of New Zealand prior to 1840 (Smith 1910)*, and *The lore of the whare-wananga* (Smith et al. 1913, 1915). Smith was awarded the Hector Memorial Medal and Prize by the New Zealand Institute in recognition of his research in Polynesian ethnology in 1920 (Tregear 1922). Byrnes notes that ‘although it is now generally accepted that much of his work on the Maori is unreliable, his research nevertheless provided a basis for the development of professional ethnology in New Zealand’ (Byrnes 2014).

**William Williams** (1800-1878), missionary and linguist, was born at Plumtre House, Nottingham, England. Initially educated at a small dame school and at Southwell Grammar School, he completed an apprenticeship as a surgeon before entering Magdalen Hall, Oxford, in 1822, as a prospective Church Missionary Society trainee. In 1824, he completed a BA in Classics, and the same year was ordained deacon and later a priest. At the beginning of 1825 he was at the Church Missionary Society Training College, Islington, London (Porter 2014). He followed his older brother, Henry, to New Zealand landing at Paihia, Bay of Islands, in 1826. At Paihia Williams was in charge of the English boys’ school and was the mission doctor. He quickly became fluent in spoken Māori and in September 1826 he began to translate the Scriptures into Māori and by the end of 1837 he had completed the whole of the New Testament and the greater part of the Book of Common Prayer (Porter 2014).

Williams made a number of missionary journeys, including a trip by schooner to the East Cape and Mahia peninsula in December 1833 and January 1834, trips overland to the Thames and Waikato regions, and a journey from East Cape to Tūranga in January 1838, with William Colenso, Richard Matthews and James Stack. On a trip to the East Coast in 1839 he agreed to take over the Waimate school and left Paihia for Tūranga on 31 December 1839. William Williams remained based at the Tūranga mission station from 20 January 1840 to 3 April 1865. After leaving Tūranga in 1865 he stayed for two years at Paihia to begin another training school at Horotutu. In May 1867, he moved to his final residence in Napier. In 1875 he established the Hukarere school for Māori girls (Porter 2014). With his many Māori converts he regularly read and conversed in Māori. His enduring memorial to Māori is *A dictionary of the New Zealand language*, first published at Paihia in 1844. He also edited the second edition, the third and fourth was edited by his son, Bishop William Leonard Williams, and the fifth, by his grandson, Bishop Herbert William Williams (Porter 2014).

**Elsdon Best** (1856 -1931), farm worker, soldier, sawmiller, health inspector, ethnographer and writer, spent much of his time interviewing Māori elders, collecting and researching Māori tribal history and lore. In 1892, Best was a foundation member of the Polynesian Society (a society that promoted the study and recording of Polynesian history and culture) and later in 1895, he joined the road-making team in Urewera district where he combined anthropological and ethnographical work with those of paymaster and storeman (Sissons 2010). While in the Urewera, Best formed a close working partnership with his key informants Tutakangahau of Maungapohatu, Paitini Wi Tapeka, Tamarau Waiari and Te Whenuanui of Tūhoe, and Hamiora Pio of Ngāti Awa, while maintaining his relationship with the Polynesian Society and the national museum. Best produced a number of important works; on various aspects of pre-European Māori life (Best 1924a), tribal history and lore of the Tūhoe tribe (Best and Board of Maori Ethnological Research (N.Z.) 1925; Best and New Zealand Dept. of Lands and Survey 1897), Māori religion and mythology (Best 1924b), Māori forest lore (Best and the New Zealand Institute 1907), the Māori school of learning (Best 1986), and Māori astronomy (Best 1922).

**Raymond Firth** (1901-2002) was an ethnologist who studied economic anthropology. In 1926 he examined the function and moral concepts associated with economic proverbs in traditional Māori society (Firth 1926) and later in 1929 he completed a doctoral thesis on the traditional economics of Māori.

Finally, a number of Māori language newspapers were published during the 19^th^ and early 20^th^ century. The first newspaper in the Māori language, *Ko te Karere o Nui Tireni*, was published by the government in 1842. It was a number of newspapers issued by the government between 1842 and 1877. Churches, philanthropists and Māori organizations also produced newspapers in Māori (Curnow 2002). In total more than 40 separate periodicals, some circulated nationally and others regionally, were published between 1842 and 1933 (Curnow et al. 2002; Griffith et al. 1997; McRae 2014). Māori-language newspaper history can be described in three phases. The first phase, beginning in 1842, saw a number of publications produced by the government for colonizing purposes. Many of the philanthropic and church newspapers also appeared during this period were ‘to a degree aligned to government, although giving a greater emphasis to scriptural and religious material’ (Curnow 2002). The second phase, from 1862, saw Māori-owned newspapers flourish. These papers were used to ‘apprise government and Pakeha of Maori opinion, to unify Maori thought and action, particularly in regard to land, and to educate Maori about their own society and world’ (Curnow 2002). The third phase, after 1913, saw fewer papers produced covering a broad range of subjects (Curnow 2002). The Māori language newspapers ‘advocated for their own viewpoints, but they also carried editorials, letters, articles, national, provincial and international news, notices, advertisements and obituaries. They offer a wide-ranging and distinct account of this period of New Zealand history, recording the interaction between Māori and [Pākehā](http://www.teara.govt.nz/en/glossary#Pākehā) in government, war, religion, education, everyday life – and newspaper publication. The papers are an especially valuable record of Māori history, covering cultural traditions, social life, political aspirations, debate over government, and tribal life’ (McRae 2014).

**Table S1.** Bird species that appear in *whakataukī*, and estimated mean weights. Weights are averaged across males and females). Weight data are drawn from Dunning (2007). Because Māori names do not always map exactly to modern taxonomic units, some bird weights are averaged across a group of related species, *e.g.*, ‘piopio’ (North Island piopio and South Island piopio), and ‘moa’ (all known species). In some cases, the mean average weight for the species that is most logically referenced in the *whakataukī* is used, *e.g.*, South Island snipe for ‘snipe’, and North Island kiwi for ‘kiwi’. ‘Albatross’ is similarly an average of mollymawks and albatrosses, and weights for the broad billed prion were used to estimate ‘petrel’ weights.

| Common name (s) | Scientific name | Weight (g) |
| --- | --- | --- |
| Albatross, toroa | *Diomedea/Thalassarche spp.* | 7650 |
| Bellbird, kōpara | *Anthornis melanura* | 27.15 |
| Black backed gull, karoro | *Larus dominicanus* | 941 |
| Bittern, matuku | *Botaurus poiciloptilus* | 1110.5 |
| Duck (grey), pārera, turuki | *Anas superciliosa superciliosa* | 1074 |
| Falcon, kāeaea, kārearea | *Falco novaeseelandiae* | 430.5 |
| Fantail, tīwaiwaka, tīwakawaka, tīrairaka | *Rhipidura fuliginosa* | 8 |
| Fernbird, mātātā | *Megalurus punctatus* | 25.2 |
| Gannet, tara | *Morus serrator* | 2350 |
| Godwit, kūaka | *Limosa lapponica* | 307 |
| Grey warbler, hōrirerire, riroriro, tāriroriro | *Gerygone igata* | 6.4 |
| Harrier hawk, kāhu | *Circus approximans* | 755 |
| Huia | *Heteralocha acutirostris* | 406 |
| Kākā | *Nestor meridionalis* | 429 |
| Kākāpō | *Strigops habroptilus* | 1750 |
| Kākāriki, porete, parakeet | *Cyanomorphus spp.* | 75 |
| Kea | *Nestor notabilis* | 800 |
| Kererū, kūkū, kūkupa, NZ woodpigeon | *Hemiphaga novaeseelandiae* | 653 |
| Kingfisher, kōtare | *Halcyon sancta vagans* | 64.2 |
| Kiwi | *Apteryx mantelli (N.I. kiwi)* | 2330 |
| Kōkako | *Callaeas cinereus (N.I. kokako)* | 65 |
| Laughing owl, hakoke | *Sceloglaux albifacies* | 600 |
| Longtailed cuckoo, koekoeā | *Eudynamys taitensis* | 117 |
| Moa, manu-whakatau, kuranui | *Dinornithidae; Dinornis* | 230000 |
| Moho, pōpōtai | *Porphyrio mantelli* | 2470.5 |
| Morepork, koukou, ruru | *Ninox novaeseelandiae* | 164 |
| Muttonbird, tītī, sooty shearwater | *Puffinus griseus* | 787 |
| Oystercatcher, tōrea | *Haematopus ostragegus finschi* | 535.5 |
| Piopio | *Turnagra turnagra (N.I. piopio)* | 196 |
| Broad-billed prion, pekehā | *Pachyptila vittata* | 200 |
| Pouākai, Haast’s eagle | *Harpagornis moorei* | 18000 |
| Pukeko, pākura | *Porphyrio porphyrio* | 988 |
| Quail, kōreke, kāreke, kōkōreke | *Coturniz novaezelandiae* | 210 |
| Robin, tōtōara | *Petroica australis* | 27.35 |
| Saddleback, tīeke | *Philesturnus carunculatus* | 74.95 |
| Silvereye, pītongatonga | *Zosterops lateralis* | 13 |
| Shag, kawau | *Phalacrocorax carbo novaehollandiae* | 2000 |
| Shining cuckoo, pipiwharauroa | *Chrysococcyx lucidas* | 24 |
| Snipe, hokio, hakuwai, hōkioi | *Coenocorypha iredalei* | 110 |
| Tomtit, miromiro, hōrimorimo | *Petroica megacephala* | 11.8 |
| Tūī, kōkō, parsonbird | *Prosthemadera novaeseelandiae* | 107.3 |
| Weka | *Gallirallus australis* | 851.5 |
| White heron, kōtuku | *Egretta alba modesta* | 950 |
| Whitehead, pōporoihewa | *Mohoua albicilla* | 16.5 |

**Table S2.** Extinct birds from the two main islands of New Zealand, estimated extinction dates, and their appearance in Māori *whakataukī*. Likely extinction period estimates are largely drawn from Tennyson (2006), Wood ( 2013), and Perry et al. (2014), based on archaeological estimates, likely functional extinction dates, and last sightings. For some species, broad estimates are given, and the median date within the likely extinction period is therefore used. In many cases, there are a number of possible Māori names, and only the most common name is shown. Māori names shown here generally accord with those that occur in *whakataukī*.

# moa are treated as one group, as the relationship between currently known names for moa and individual species is unclear.

* hokioi is recorded here as a variant of hakuwai, meaning snipe, as in our view this is most likely to be the context for its occurrence in the ancestral sayings that we examined here; however, there is a broader debate about its usage, particularly whether hokioi is another term for the pouākai, Haast’s eagle, as in some narratives, for example.

** piopio is listed for both North Island (NI) and South Island (SI) species, although it may be that piopio is a South Island term, with a separate name known for the North Island piopio (P. Scofield, pers. comm.).

+ black swan became extinct after Polynesian arrival, before reinvading from Australia.

++ possible sightings were made of the owlet nightjar as late as the 19^th^ century, but other evidence suggests it may have become extinct not long after Polynesian arrival; hence no date is provided here.

| **Order** | **Family** | **Scientific name** | **Common name** | **Common Māori name** | **Likely extinction period** | **Number of *whakataukī*** |
| --- | --- | --- | --- | --- | --- | --- |
| Dinornithiformes | Emeidae | *Megalapteryx didinus* | Upland moa | moa | 1450 | 30^#^ |
| Dinornithiformes | Dinornithidae | *Dinornis novaezelandiae* | NI giant moa | moa | 1450 |  |
| Dinornithiformes | Dinornithidae | *Dinornis robustus* | SI giant moa | moa | 1450 |  |
| Dinornithiformes | Emeidae | *Pachyornis australis* | Crested moa | moa | 1450 |  |
| Dinornithiformes | Emeidae | *Pachyornis elephantopus* | Heavy footed moa | moa | 1450 |  |
| Dinornithiformes | Emeidae | *Pachyornis geranoides* | Mantell’s moa | moa | 1450 |  |
| Dinornithiformes | Emeidae | *Anomalopteryx didiformis* | Little bush moa | moa | 1450 |  |
| Dinornithiformes | Emeidae | *Emeus crassus* | Eastern moa | moa | 1450 |  |
| Dinornithiformes | Emeidae | *Eurapteryx curtus* | Stout legged moa | moa | 1450 |  |
| Gruiformes | Aptornithidae | *Aptornis otidiformes* | NI adzebill |  | 1450 | 0 |
| Gruiformes | Aptornithidae | *Aptornis defossor* | SI adzebill |  | 1450 | 0 |
| Anseriformes | Anatidae | *Cnemiornis calcitrans* | SI goose |  | 1450 | 0 |
| Anseriformes | Anatidae | *Cnemiornis gracilis* | NI goose |  | 1450 | 0 |
| Accipitriformes | Accipitridae | *Aquila moorei* | Haast’s eagle | pouākai | 1500 | 1 |
| Anseriformes | Anatidae | *Oxyura vantetsi* | Stiff tailed duck |  | 1550 | 0 |
| Anseriformes | Anatidae | *Biziura delautouri* | Musk duck |  | 1550 | 0 |
| Accipitriformes | Accipitridae | *Circus teauteensis* | Forbes’ harrier |  | 1550 | 0 |
| Gruiformes | Rallidae | *Capellirallus karamu* | Snipe rail |  | 1550 | 0 |
| Gruiformes | Rallidae | *Fulica prisca* | NZ coot |  | 1550 | 0 |
| Passeriformes | Acanthisittidae | *Dendroscansor decurvirostris* | Long billed wren |  | 1550 | 0 |
| Passeriformes | Acanthisittidae | *Pachyplichas yaldwyni* | Stout legged wren |  | 1550 | 0 |
| Passeriformes | Corvidae | *Corvus antipodum* | NZ raven |  | 1550 | 0 |
| Anseriformes | Anatidae | *Chenonetta finschi* | Finsch’s duck |  | 1600 | 0 |
| Anseriformes | Anatidae | *Malacorhynchus scarletti* | Scarlett’s duck |  | 1600 | 0 |
| Procellariiformes | Procellariidae | *Puffinus spelaeus* | Scarlett’s shearwater |  | 1662 | 0 |
| Gruiformes | Rallidae | *Gallinula hodgenorum* | Hodgen’s waterhen |  | 1750 | 0 |
| Anseriformes | Anatidae | *Mergus australis* | Southern merganser |  |  | 0 |
| Sphenisciformes | Spheniscidae | *Megadyptes waitaha* | Waitaha penguin |  | 1500 | 0 |
| Anseriformes | Anatidae | *Cygnus atratus* | Black swan |  | + | 0 |
| Caprimulgiformes | Aegothelidae | *Aegotheles novaezelandiae* | NZ owlet nightjar |  | ++ | 0 |
| Galliformes | Phasianidae | *Coturnix novaezelandiae* | NZ quail | koreke | 1875 | 4 |
| Charadriiformes | Scolopacidae | *Coenocorypha barrierensis* | NI snipe | hakuwai, hokioi* | 1875 | 5 |
| Gruiformes | Rallidae | *Porphyrio mantelli* | NI takahe | moho | 1894 | 7 |
| Ciconiiformes | Ardeidae | *Ixobrychus novaezelandiae* | NZ little bittern | kaoriki | 1890 | 0 |
| Passeriformes | Acanthisittidae | *Traversia lyalli* | Lyall’s wren |  | 1895 | 0 |
| Passeriformes | Turnagridae | *Turnagra tanagra* | NI piopio | piopio** | 1902 |  |
| Passeriformes | Turnagridae | *Turnagra capensis* | SI piopio | piopio | 1905 | 1 |
| Strigiformes | Strigidae | *Sceloglaux albifacies* | Laughing owl | whekau | 1914 | 1 |
| Passeriformes | Callaeidae | *Heteralocha acutirostris* | Huia | huia | 1925 | 2 |
| Charadriiformes | Scolopacidae | *Coenocorypha iredalei* | SI snipe | tutukiwi | 1964 | 0 |
| Passeriformes | Acanthisittidae | *Xenicus longipes* | Bush wren | mātuhi | 1972 | 0 |

**Table S3.** Examples of biological observations in *whakataukī.*

| ***Whakataukī*** | **English translation** | **Ecological context** | **Māori context^1^** | **Time period** |
| --- | --- | --- | --- | --- |
| Me te tarakihi e pāpā ana i te waru | Like the cicada chirping in the eighth month | Cicada chirping is common in the eight month of the Māori year (summer) | A comparison with animated human conversation | 1350-1500 |
| Me he toroa ngunungunu | Like an albatross crouching | Describes the action of an albatross neatly folding its wings when coming to rest on water | Applied to the tidy folding of mats and garments | 1350-1500 |
| He tītī whāngaia tahi | The bird of a single feeding | During the day, adult sooty shearwater leave their chicks in the burrows to forage for food at sea, returning just after dusk to feed them | The saying can be applied to someone who eats sparingly | 1350-1500 |
| Pārera apu paru | The grey duck crams mud into its bill | The grey duck sometimes brings up mud with the weeds it feeds on, leading to the supposition that the grey duck’s diet included mud | A person’s diet has an effect on their health | 1350-1500 |
| Kua kitea a Matariki, ā, kua maoka te hinu | When the Pleiades are first seen at the time of sunrise, the fat is cooked | The rise of the constellation Pleiades on the horizon in June/July was a time when the NZ pigeon or kererū were taken in large numbers. These birds were then preserved in their own fat, so that the seal was airtight, and then stored to be eaten at a later date. In May and June, the kererū chiefly feeds on the miro (*Prumnopitys ferruginea*) and pate (*Schefflera digitata*) trees. At this time, it reaches its prime and it is much sought after | The reference to fat refers to the heating of fat to preserve traditional Māori delicacies, such as rats and birds. The rising of Pleiades also marked the beginning of the Māori new year | 1500-1650 |
| He tūngoungou ki te one, puta ki te ao he hīhue | A pupa in the earth emerges to be the sphinx moth | A tūngoungou, the pupa form of the hawk moth (*Agrius convolvuli*), feeds on *Calystegia* (convolvulus) species and on the highly valued cultivated kūmara (*Ipomoea batatas*) plants. It is thus an unwanted guest. The pupa lives in rotten wood, before emerging as a grey-brown native moth called hīhue in Māori. When it first emerges, its appearance is dark | This metaphor is applied to a rather ordinary person who appears on the marae in fine clothing as a chiefly orator but has nothing of substance to offer | 1500-1650 |
| Kia mate ā-Ururoa! Kei mate tarakihi! | Die like shark, not like tarakihi | Here the shark, paragon of courage and determination, is personified as the apex predator and difficult to catch. However, the tarakihi (*Nemadactylus macropterus*) is easily caught and succumbs quickly without a struggle | The shark was admired for fighting to the end. In times of war, it was considered better to die fighting than to give up easily. This example describes an action and its intended outcome |  |
| Ko te kūkū horo tāepa | The pigeon eats till it falls down | When the drupes of the miro tree are ripe the native pigeon or kererū (*Hemiphaga novaeseelandiae*) will gorge itself until it is unable to perch securely. Miro fruit on masse in Autumn (May and June), and are a major of food for kererū. Berries can ferment in the crop of the kererū, causing inebriation |  | 1650-1800 |
| Honoa te hono a te kiore | Follow each other like rats | Rats follow close behind one another as they coursed over forest trails at night | This was advice given to warriors as they advanced to attack to ensure they were close enough to assist one another | 1650-1800 |

^1.^ Explanations based on those of Mead and Grove (2001), with additional ecological notes.

**Table S4.** Randomized sentences, with 100 examples from each of two time periods (1940s and 2010s). Sentence order, speaker details, source, and date of the interview or recording is provided.

| **Order** | **Sentence** | **Name** | **Source** | **Date** |
| --- | --- | --- | --- | --- |
| 1 | Mai i te wāhi ka hoko ai te ika, ā tae noa ki te wāhi i huti ai i ngā wai ngā moana o Tangaroa, kia pai te tuitui mai i te tīmatatanga ki te wāhi i mutu ai. | Whaimutu Dewes | Te Karere | Dec 12, 2014 |
| 2 | Kore rawa e mōhiotia, e pēhea te mahitanga ake e kai ana mai i te manawa o te Pākehā. | Te Kapa Pōtae | Mobile Unit | 1947 |
| 3 | Ngā wawata me ngā tūmanako o tātau rangatahi pakeke huri noa i te hapori katoa, koia nā te tino wawata o tātou i roto i ngā maramara. | Leon Wharekura | Te Karere | Dec 2, 2014 |
| 4 | Ko tāku ki ngā minita katoa me titiro ki te whānau kaua ko te tamaiti tōna kotahi hei te mea pā ki tētahi ka pā ki te whānau nē, tērā āhuatanga. | Te Ururoa Flavell | Te Karere | Dec 1, 2014 |
| 5 | Me te kata mai ki te koroua e noho atu rā e tūturi ki runga i ana pona me tana taiaha. | Te Kapa Pōtae | Mobile Unit | 1947 |
| 6 | I whakatutukitia ai tērā kaupapa na te mea i tērā wā ētahi reo me ētahi kanohi i oti i a rātou te kite he aha ngā hua pai rawa noa atu. | Shane Jones | Te Karere | Dec 3, 2014 |
| 7 | Ka haere tonu mai ngā whānau o Ngāti Whakaue tukuna mai ngā mokopuna ki konei ki te ako i reo o Tūhoe, ā te wā ka whakatika te mita ki reo o Ngāti Whakaue. | Colin Bennett | Te Karere | Dec 1, 2014 |
| 8 | Mehemea ka titiro te kaiwhakawā ki te mana ki te tika ki te hē o ngā taha e rua, mōku ake ko te hua he tika. | Wi Pere Mita | Te Karere | Dec 11, 2014 |
| 9 | Nō te taenga ki te raumati ka mahana te kiri o te tangata kātahi anō ka whakahaua e Turi ngā tamariki kia haere ki te kaukau i Waimatuhirangi. | Māui Onekura | Mobile Unit | 1946 |
| 10 | Ia tau ia tau ka haere mātou te whānau o Kahakura ki te tautoko o tērā ko te tūmanako ka haere mai ngā tamariki o Hone Waititi. | Tania Mahuru-Stanley | Te Karere | Dec 11, 2014 |
| 11 | Nā Te Ua Haumene te pakanga tuatahi, te Pākehā, i tēnei motu, pakangatia ai i te Pākehā, ko Wairau, nā Te Haumene. | Toroa Ngatau | Mobile Unit | 1946 |
| 12 | Nā, te ingoa hapū e karangatia ana ko ngā uri o Tamatepō ko Teuringahu o Ngāti Rongoū. | Toki Watene | Mobile Unit | 1947 |
| 13 | Ko te pūtake i whakataua i tēnei hui me mutu te whawhai me whakatupu te rākau o te maungārongo ki runga i te motu hei marumaru mō ngā iwi e rua Pākehā me ngā Māori. | Raureti Te Huia | Mobile Unit | 1947 |
| 14 | Ka pōhiritia ki uta, ka tae te tukuna a Paora Te Putu te moana me te whenua ki a Ngāti Porou. | Te Kapa Pōtae | Mobile Unit | 1947 |
| 15 | Kāore whakarongo kāore taringa he hiahia ki te kōrero tika kōrero pono ki a mātou te whānau me ngā hapū. | Rueben Taipari Porter | Te Karere | Dec 10, 2014 |
| 16 | Ko ngā pātai i tukua atu i te wiki i pahure ake nei heoi kei matuku ahau kei ngaro te ia o taku pātai. | Peeni Henare | Te Karere | Dec 9, 2014 |
| 17 | Kaua ko Ngāi Māori anake kua uru mai ngā kīwaha ngā wheako o te ao Māori ki roto o ngā rotarota hei whakarata ake. | Te Utanga Tautuhi | Te Karere | Dec 4, 2014 |
| 18 | Nā ōna mahi kino koia te take i patua ai a ia, ā, waiho ana hei kino mō ōna uri, arā, mō Ngāti Kōtare me Ngāti Kauwhata. | Raureti Te Huia | Mobile Unit | 1947 |
| 19 | Ko te mea nā kei te whakamataku i a mātou na te mea kua tino wikitōria mātou kāore e kore ka takahuiri te karauna ki te turaki i a mātou tō mātou nei ture. | Maanu Paul | Te Karere | Dec 5, 2014 |
| 20 | Ki te mahi ai i ngā mahi a ō mātou nei tīpuna me kī te whakatere i ēnei o ngā waka haurua ki runga i te moana. | Rereahu Hetet | Te Karere | Dec 5, 2014 |
| 21 | Ki tā ngā kaumātua kōrero, ko te tīmatanga o aua rangi e tīmata mai ana i a Whiro e mutu ana i a Mutuwhenua. | Raureti Te Huia | Mobile Unit | 1947 |
| 22 | Ko te take i takotongia ai tēnei kupu, tēnei kupu a Paora Te Putu ki runga i ngā rangatira o Hauraki. | Te Kapa Pōtae | Mobile Unit | 1947 |
| 23 | I roto i ngā tau, ko te Karauna kāore i tika te mahi pērā hoki rā i te awa o Waikato. | Rahui Papa | Te Karere | Dec 10, 2014 |
| 24 | Ko te tikanga ki tō mātou kura kei a mātou he momo puna wai hei parakatihi i a rātou ki ēnei momo mahi. | Sam Iraia | Te Karere | Dec 4, 2014 |
| 25 | Te mahi i mahingia e ngā waka nei pātere ana ngā hinu me ngā paitini kei roto i ō mātou one. | Bundy Waitai | Te Karere | Dec 16, 2014 |
| 26 | Raweke tātou e tae ana ki te teiteitanga o te kaumātuatanga kua matemate i ō tātou whanaunga. | Iwipuihi Percy Tipene | Te Karere | Dec 17, 2014 |
| 27 | He hēmana ia mō te rūnanga he rōpū mō te Kawana, tērā te rūnanga o Te Rarawa ehara mō mātou te whānau me te hapū | Rueben Taipari Porter | Te Karere | Dec 10, 2014 |
| 28 | Kua hui mātou ko te hāhi i runga i ēnā kaupapa, ā te hanga mahere pea me pēhea mātou e hanga whakamua. | John Mathews | Te Karere | Dec 2, 2014 |
| 29 | Ko au e mea ana, hei aha tēnā wāhanga i roto i te karakia anō, ā me te mea nei ka tae ki te nuinga o te inoi kua makere mai te ingoa o Ihu Karaiti i te āhua karaitiana i roto i te karakia. | Pihopa Kitohi Pikaahu | Te Karere | Dec 10, 2014 |
| 30 | Nā, ka kitea e Turi te whakaaro me rapu e ia ki a Hawepōtiki, ki te tamaiti a Uenuku. | Māui Onekura | Mobile Unit | 1946 |
| 31 | Ka moe tā Hako, ko Te Karu o te Rangi ka moe i a Pūawa, kia puta tonu mai ki waho ko Ruawehea, ka moe i a Tamaterā. | Hoani Te Huia | Mobile Unit | 1947 |
| 32 | Nō reira, ki te mōhio ia ki ēnei tūtuku me tēnei karakia me te karo kua wātea ia i runga i ngā patu a tētehi iwi kē ā ētehi atu āhua tāngata mākutu. | Raureti Te Huia | Mobile Unit | 1947 |
| 33 | Ki te kore te Kāhui Minita e mōhio ka pēhea te āhuatanga o te Whānau Ora ka kore rātou e tuku pūtea mai ki te āwhina ki te tautoko i te hurahi e whaiwhai haeretia ana nei e te Whānau Ora. | Te Ururoa Flavell | Te Karere | Dec 1, 2014 |
| 34 | Arā, he rīhi i te wā i pānuitia mai ai te tīti ki te hunga nō rātou te whenua. | Raureti Te Huia | Mobile Unit | 1947 |
| 35 | He kōrero pono tērā i te mea i ngā wā koia nā te ingoa o tēnei rohe o tātou ko Rahui Pokeka tērā. | Norman Hill | Te Karere | Dec 2, 2014 |
| 36 | Taihoa pea kia kite ka pēhea rā te whakatakoto a te motu ōna whakaaro, engari ko tāku e tūmanako nei ka whakatinanahia tā te motu e whakatau ai i tēnei rā nei. | Jeremy Tātere-McLeod | Te Karere | Dec 11, 2014 |
| 37 | Ināianei nā ka kitea atu te tikanga i ā rātou nei mahi i tērā wā kia wātea hoki rātou te tuku atu i wāhi ki ngā kamupene. | Haami Piripi | Te Karere | Dec 9, 2014 |
| 38 | Kei te tino ohorere au ki te nui o ngā mea pai kua pā mai ki a au i ngā rā e rua kua pahemo. | Apirana Pewhairangi | Te Karere | Nov 30, 2014 |
| 39 | Koia nei tāku ki tā te Minita o te Mātauranga anō nā ki a rātou o te Kōhanga reo kei konei au te tautoko te āwhina i a rātou ina hiahia. | Marama Fox | Te Karere | Dec 11, 2014 |
| 40 | Arā, ngā uri o Te Ngako e kīa ana ko Ngāti Maru tūturu tēnā i runga i te mea koinā te huarahi tāne o tēnei karangatanga o Ngāti Maru | Toki Watene | Mobile Unit | 1947 |
| 41 | Nā te mea kei te mataku mātou ina ka haere mai wētahi ki te tūkino i ō mātou whare tūkino i ō mātou kaikarakia tūkino i ā mātou tamariki ā mātou wāhine. | Te Rata Hikairo | Te Karere | Dec 16, 2014 |
| 42 | E Turi, tēnei te maire i rongo atu ai au kei te waiatatia mai i roto i Wharekura. | Māui Onekura | Mobile Unit | 1946 |
| 43 | Kua whakapono mai te tianara ki ā mātou kōrero kua whuia ngā kōrero a te karauna ki tahaki ana he mea nui tēnā. | Maanu Paul | Te Karere | Dec 5, 2014 |
| 44 | Nō te wā o te whakatupuranga a Tāwhao me te whakatupuranga a Tuhianga ka tīmata ngā raruraru ki Kāwhia ki waenganui i a Tainui. | Raureti Te Huia | Mobile Unit | 1947 |
| 45 | Te mutunga ake o ngā mahi toi nei, ko te tūmanako kia uru atu ki ngā mahi tiaki taonga ki ngā whare taonga. | Te Utanga Tautuhi | Te Karere | Dec 4, 2014 |
| 46 | Āe na te mea i te kāinga ki āku nei whakaaro he puhi ahau he kuini rānei nō reira ae kāore au i tino mōhio me aha mō tēnei kiriata engari he whāinga mōku. | Moana Te Hei | Te Karere | Dec 3, 2014 |
| 47 | I reira ka haere mai ngā uri ka torotoro mai ki tēnei taha, arā, ki tētehi pā ko Ōruarangi i reira, ko Ōruarangi. | Toki Watene | Mobile Unit | 1947 |
| 48 | Ko Te Orangi kei te puru i te pū, te matā, te paura, ka mutu, ka hoake tana hoa ki a Wiremu Kīngi Matakātea mā tērā e pupuhi. | Toroa Ngatau | Mobile Unit | 1946 |
| 49 | Ē, i roto i ngā kōrero a ngā kaumātua, ā, nā i roto i ngā whare kura e toru ngā wāhanga o te tau. | Raureti Te Huia | Mobile Unit | 1947 |
| 50 | He kupu whakamaharatanga tēnei mō ngā āhuatanga o ngā tūpuna i te wā whakaririkatia ai e rātou. | Toroa Ngatau | Mobile Unit | 1946 |
| 51 | Ka kaha te haere o te hāhi o ngā iwi ki roto ki te hāhi Katorika, ka puta te pūhaehae. | Toroa Ngatau | Mobile Unit | 1946 |
| 52 | Kua kite au i a Rocky, tahi tau kua noho ia ki konei ki tōku nei taha, ā kua piki nei tāna taumata tana mōhiotanga ki tāna reo ake. | Sheree Waitoa | Te Karere | Dec 2, 2014 |
| 53 | Ko ngā pukapuka i kohia mai e taua hui he mea tuhituhi nā Hīti Paiariki te tau kotahi mano e waru rau e waru tekau mā whā i tuhituhi ai ia ki Makōkōmiko. | Raureti Te Huia | Mobile Unit | 1947 |
| 54 | Tēnā mea te hua parakore he kai kāhore i uru anō tēnā mea te paihana kāhore anō i raweke anō te ira o ngā kākano mai rāno | Iwipuihi Percy Tipene | Te Karere | Dec 17, 2014 |
| 55 | Ko ngā mahi a ngā tāngata i te ngahuru, ē, he haere he kimi kai he mahi manu i ngā wā e tika ana. | Raureti Te Huia | Mobile Unit | 1947 |
| 56 | Otirā, nā te hara o tōna hoa wahine ki tā rāua mōkai, ki a Rotu, koia ka riro te mana o Tainui ki a Hotu-roa. | Raureti Te Huia | Mobile Unit | 1947 |
| 57 | Te tangata e kīa nei o Ngāti Maru o roto o Hauraki, arā, ka moe a Marutūahu ka moea ngā wāhine. | Toki Watene | Mobile Unit | 1947 |
| 58 | Ina whiwhia au i te tūranga ina kāore au i whiawhia he mea nui tēnei mōku. | Moana Te Hei | Te Karere | Dec 3, 2014 |
| 59 | He mīhini te reo Māori ko te mea tuatahi he mīhini pūtea mō tātou te whānau i runga i te marae. | Maria Kapa-Kingi | Te Karere | Dec 5, 2014 |
| 60 | Korekau he tūru Māori i roto i te kaunihera, ae he poari Māori e noho tata ki te kaunihera engari kāore he mana tā te rōpū kia taunaki motuhake ētahi whakaaro. | Marama Fox | Te Karere | Dec 3, 2014 |
| 61 | Ko te wāhi e kōrerotia ake nei ināianei ko te wāhi e pā ana mō tēnei wāhi e karangatia neki ko Ruawehea. | Hoani Te Huia | Mobile Unit | 1947 |
| 62 | Mena koia rā te ara e pīrangi ana ngā mātua te whai ō rātou tamariki ki te whai, anā koia nei anake te kōwhiringa ki te tēnei takiwā. | Kanapu Rangitauira | Te Karere | Dec 12, 2014 |
| 63 | Tae mai te tangata rā a Mōhi Mangakāhia, kātahi ka tū ki runga, whaikōrero ki a Ngāti Porou, ki ngā iwi katoa. | Te Kapa Pōtae | Mobile Unit | 1947 |
| 64 | Kātahi ka rongohia atu, e Rongorongo ka mōhio a Turi kātahi ka mea atu ki a Rongorongo, kua mōhio e Turi, arā, kua mōhio e Turi. | Māui Onekura | Mobile Unit | 1946 |
| 65 | Ko tāku e tautokongia ana i tēnei āhuatanga e taea ana ngā taha e rua kia whakaputa ō rātou ake whakaaro e pā ana tēnei kaupapa ngā kereme o Ngāpuhi. | Kelvin Davis | Te Karere | Dec 3, 2014 |
| 66 | Tērā pea koia nā tētahi o ngā pātai ka whakatakotohia ki mua te aro o tēnei rōpū mā rātou anō rā tērā e whakautu ka whakahoki kōrero mai ki a au ka āta titirohia i tērā wā. | Te Ururoa Flavell | Te Karere | Dec 4, 2014 |
| 67 | Kei raro iho hoki i taua puke i tū te whare karakia tuatahi a Te Kooti, he hāhi Ringatū neki tana hāhi. | Hoani Te Huia | Mobile Unit | 1947 |
| 68 | Nō reira tana mōhiotanga mea ake ia whakamatea hei utu mō taua tamaiti a Uenuku. | Māui Onekura | Mobile Unit | 1946 |
| 69 | I kore e āhei ki te haere ki ngā parakatitihi nā reira, kei te āhua mokemoke nō te mea ko tāku whānau tērā kei te haka, ko ahau kei te whai atu i tētahi atu rā. | Rob Ruha | Te Karere | Dec 10, 2014 |
| 70 | Kia kaua hei whakamā, mēnā e mōhio ana rātou ētahi kupu ētahi o ngā waiata me tūtū mai ki te waiata i ō mātou nei taha. | Maisey Rika | Te Karere | Dec 11, 2014 |
| 71 | Koinā te pūtake o tēnā waiata o ngā rarurarutanga i reira i taua wā mō ngā mahi a aua tohunga. | Raureti Te Huia | Mobile Unit | 1947 |
| 72 | Heoi ka mutu i konei ngā tātai kōrero i tīmataria ai te noho e ngā tūpuna ō rātou ora ō rātou mate. | Raureti Te Huia | Mobile Unit | 1947 |
| 73 | Pērā i te hitori o ia kura he nui ngā aupiki me ngā auheke koia rā te āhuatanga o ēnei momo kura ka kaha pehia e te ao Pākehā. | Kanapu Rangitauira | Te Karere | Dec 12, 2014 |
| 74 | Nā Rapata Wahawaha kua oti, ka whiriwhiritia ngā tāngata kua oti te mārama o Te Aowera, Te Whānau a Rakairoa. | Te Kapa Pōtae | Mobile Unit | 1947 |
| 75 | I a ia ka whakareri,whakarite mō te haere mai, ka kīia mai ia e ngā tāngata o Hawaiki, arā, te tangata o runga i taua waka, a Hotunui. | Toki Watene | Mobile Unit | 1947 |
| 76 | Pānui i ngā tai, te āhua o Tawhirimātea i wānanga mātou e pā ana ki ngā whetū, i tu atu i tērā ka pēhea te haumarutanga. | Tania Mahuru-Stanley | Te Karere | Dec 11, 2014 |
| 77 | E kore au te mōhio ngā tīpuna kua wehea mai nei i te pāpā i whānau ai rātou i tipu ake rātou. | Pou Temara | Te Karere | Dec 1, 2014 |
| 78 | Ko tā rātou whakawā mō ēnei take kino he taua tukutuku maru, arā, he tono kia utua te hara. | Raureti Te Huia | Mobile Unit | 1947 |
| 79 | Nā Ngāpuhi te kau i tō mai riro kē mai ētahi atu i miraka i te tuatahi nā reira kua roa kē te tata mutu te whakatau i ngā kereme he aha ai i waihotia ai a Ngāpuhi mō te mea mutunga. | Erima Henare | Te Karere | Nov 30, 2014 |
| 80 | He kaupapa whakahirahira tēnei, ehara i te mea mō Te Papa anake, kāo mō tātou katoa. | Arapata Hakiwai | Te Karere | Dec 4, 2014 |
| 81 | Ko te tino kaupapa me korero tahi matou o te hau kainga ki tērā o ngā taumata o te kaunihera ra no reira koia to matou | Norman Hill | Te Karere | Dec 2, 2014 |
| 82 | I pātai te pātai i roto i te reo Pākehā i runga i te mōhio ehara tēnei hei wero atu mō tōna hiahia kia ora ai te reo. | Nanaia Mahuta | Te Karere | Dec 9, 2014 |
| 83 | Kei te titiro au i te tuku ihotanga o te reo i roto i ngā whānau, nā reira mai i ngā pakeke mai i ngā mātua ki ngā tamariki ki ngā mokopuna i te mea ki a au nei kei reira te oranga tonutanga o te reo. | Te Kuru Dewes | Te Karere | Dec 11, 2014 |
| 84 | Kei te kitea i tēnei wā ngā hua puta mai i ngā mahi ohaoha kua oti te mahi, ā ki ngā mea kei mua tonu i a tātou. | Whaimutu Dewes | Te Karere | Dec 12, 2014 |
| 85 | Ko te tikanga ia ka whakakotahi nei ngā kōrero e rua nei mō te painga o te piri, otirā mō te pai o tō tātou reo nei i tōna mutunga. | Te Ururoa Flavell | Te Karere | Dec 4, 2014 |
| 86 | Ki te whakahou anō te inoi me whakatika anō i ngā kupu kei roto i te reo Pākehā, ngā kupu o te wā o ō tātou mātua. | Pihopa Kitohi Pikaahu | Te Karere | Dec 10, 2014 |
| 87 | Ko tōna tīmatanga mai i muri iho tēnei kōhanga i tētahi whare hāhi i reira mātou e mahi ana i ngā mahi o te kōhanga. | Taumata Solomon | Te Karere | Dec 1, 2014 |
| 88 | Te kaiwhakaoti ēnei rarurarutanga ka pā, nā Te Ua Haumene rāua ko Te Whiti o Rongomai te tatūtanga mai o te Kīngi Tāwhiao, i Meremere. | Toroa Ngatau | Mobile Unit | 1946 |
| 89 | Koia nā te take ka karangatia te ingoa o Te Orangi ko ngā tae rākau nui nā taua kupu whakawai a Waikato mōna. | Toroa Ngatau | Mobile Unit | 1946 |
| 90 | Ko te ingoa o tēnei hapū i mua atu i a Paikea ko Te Whānau a Kupe. | Te Kapa Pōtae | Mobile Unit | 1947 |
| 91 | Pukerangiora te pā pakangatia ai i tērā pakanga, tērā wā muri mai, ngā pakanga muri mai, tae mai te pakanga ki Te Namu. | Toroa Ngatau | Mobile Unit | 1946 |
| 92 | Ko ia te manu e rērere haere ana i ngā teitei o te pū whakaaro, te tangata e tino matatau ana ki tēnei mahi ki te mahi makaurangi ki te mahi kōwhaiwhai, koia tāna. | Derek Lardelli | Te Karere | Nov 30, 2014 |
| 93 | Nā te tīhorehoretanga o ngā rerekētanga o te hapū o te whānau koia ia tēnā te take i tino ngawari nei te ngau mai o tēnei nama i runga i a mātou. | Dover Samuels | Te Karere | Dec 5, 2014 |
| 94 | I hoki anō ēnei uri ki te moe i ngā uri o Whatihua rāua ko Ruapūtahanga. | Raureti Te Huia | Mobile Unit | 1947 |
| 95 | Anā ki ngā korero a tōku tipuna ko te reo te pūtake o te Māoritanga he taonga tuku iho nā ngā atua. | Te Kuru Dewes | Te Karere | Dec 11, 2014 |
| 96 | Ka ahiahi ka puta atu a Rongorongo ki waho ki te whāngai i a Tāneroroa. | Māui Onekura | Mobile Unit | 1946 |
| 97 | Mō ngā taima e rima ka tū au ki roto i te whare, ana ko aua taima ki roto i te taima e whā ka kōrero ahau ki te reo Māori. | Peeni Henare | Te Karere | Dec 9, 2014 |
| 98 | Āhua ohorere ana mātou nā te mea ngarongaro haere ngā tāngata nō reira e tika ana me kohi kōrero i te wā e ora ana, ā me kohi kōrero mō ngā uri kei te haere mai. | Mina Pomare-Peita | Te Karere | Dec 1, 2014 |
| 99 | Kua oti i a Tauranga Moana me Mataatua te whiriwhiri ō rātou whakaaro, engari kātahi anō ka kite i ngā putanga mai o ngā kōrero a te rōpū nei a te Tuara. | Huata Palmer | Te Karere | Dec 11, 2014 |
| 100 | Ka hoatu e au ngā kī o te rangatiratanga o te rangi ki a koe ko tāua paihere ai i te whenua, paihere hoki ahau i te rangi. | Toroa Ngatau | Mobile Unit | 1946 |
| 101 | Ka mau au i te wero i te manuka kua takotoria e te kaumatua rā. | Peeni Henare | Te Karere | Dec 9, 2014 |
| 102 | Te ūnga atu ki reira, ka whānau te tamaiti a te wahine nei e kīa ana ko tōna ingoa ko Paikea. | Te Kapa Pōtae | Mobile Unit | 1947 |
| 103 | I āta oma mai i ngā puke me ngā maunga kia eke rawa ki ngā kōtihitihi o ngā maunga e hiahia ana nei e au. | Apirana Pewhairangi | Te Karere | Nov 30, 2014 |
| 104 | Ō matou whare he whare tawhito kua roa mātou i noho nei ki tēnei pito o Tamaki, ā nō reira e tika ana me houhoungia ō tātou whare. | John Mathews | Te Karere | Dec 2, 2014 |
| 105 | Ko tēnei tangata ko Tekītae ko ia te rangatira nunui o Ngāti Whanaunga, ko tētahi o ōna ingoa ko Te Taniwha. | Te Kapa Pōtae | Mobile Unit | 1947 |
| 106 | Te waimarietanga kua puta mai a Tamanuiterā i te rā nei kāore anō kei te karikari ngā wai, he āhua marino te wai ināianei, nō reira he pai. | Sam Iraia | Te Karere | Dec 4, 2014 |
| 107 | Ko te mana nui o Maungatautari ko te mana nui o Waikato ko te mana nui o ngā mānia whenua o tō mātou nei rohe koia tērā e whakawhāititanga iho o te kaupapa. | Rāhui Papa | Te Karere | Dec 10, 2014 |
| 108 | Koia tēnā te whakapai, tēnā pea a te wā ka hau mai te ture ki te tango i te whenua katoa. | Dover Samuels | Te Karere | Dec 5, 2014 |
| 109 | Ko tēnei te whakahokinga mai kōiwi nui katoa mai ka tīmata mai i tēnei mahi. | Pou Temara | Te Karere | Dec 4, 2014 |
| 110 | Koia mātou e mea ai 17 o ngā tūru 22 ki runga o Tūhoronuku he tūru hapū kē. | Sonny Tau | Te Karere | Nov 30, 2014 |
| 111 | Me whakakore a Tūhoronuku, tīmata anō a Ngāpuhi ki te huihui ki te kōrero tahi ā muri i ngā kerēme. | Patu Hohepa | Te Karere | Dec 4, 2014 |
| 112 | Ko tētehi wāhanga o rātou i ahu mai i a Kurangautuku, i tēnā iwi i kīia nei he māeroero. | Raureti Te Huia | Mobile Unit | 1947 |
| 113 | Mehemea kāore i pērā ngā whakaaro a ngā kaitono, e taea ana e rātou te tono ki ngā Koti Teitei ki te Koti Pira rānei. | Wi Pere Mita | Te Karere | Dec 11, 2014 |
| 114 | Ko te painga mō te wā roa ka taea ngā tāngata katoa te whiwhi mahi kia taea e rātou te hanga he huarahi mō tō rātou whānau. | Hekia Parata | Te Karere | Dec 1, 2014 |
| 115 | Ka tae ki te wāhi te kaha pea o te mākū i ōna aituā tonu rānei. | Raureti Te Huia | Mobile Unit | 1947 |
| 116 | Haere ngā wāhine kāre au e mōhio te take tonoa rātou e Paikea ki te haere ki te tiki i te rākau nei. | Te Kapa Pōtae | Mobile Unit | 1947 |
| 117 | Nā, ka noho ngā uri i reira tae noa ki te haerenga mai ka noho ngā uri i reira | Toki Watene | Mobile Unit | 1947 |
| 118 | Kei te whakaatu ki a koutou e te whānau mā, ko te wāhi tēnei i tīmata ai. | Māui Onekura | Mobile Unit | 1946 |
| 119 | Te mea e kitea ana e rāua ko tō rātou teina ko Paikea he atua taua tangata. | Te Kapa Pōtae | Mobile Unit | 1947 |
| 120 | Kore e kore mārika e kite tēnei tūmomo rauemi, na te mea i hāngai mo te Warawara anake. | Diedre Wijohn | Te Karere | Dec 1, 2014 |
| 121 | Te poto rawa ki te whakaaro ka āmene mai tātou, mā te hui mā Te Tai Rāwhiti e whakamana ehara mā te kohi nei. | Kohi Coleman | Te Karere | Dec 11, 2014 |
| 122 | Nā, ka tapaina tēnā wāhi mai i taua rā ki tēnei rā ko Whitianga, nā Paikea. | Te Kapa Pōtae | Mobile Unit | 1947 |
| 123 | Ko te ao o te waka he momo ao hei wero i te hinengaro hei wero i te tinana hei wero i te wairua. | Hoturoa Kerr | Te Karere | Dec 5, 2014 |
| 124 | I tū ai tētahi haki he haki pango ko aua kupu tapu anō kei runga i taua haki. | Te Rata Hikairo | Te Karere | Dec 16, 2014 |
| 125 | Ka tono a ia ka hōmai e Te Taniwha, ko Manaia, e rima mano eka, ki a Te Kohu-o-Rehua me tana iwi. | Te Kapa Pōtae | Mobile Unit | 1947 |
| 126 | Ka tapaina te ingoa o tēnā maunga mai i taua rā ki tēnei rā ko Pāuanui. | Te Kapa Pōtae | Mobile Unit | 1947 |
| 127 | Ki te tiro au ki ngā pēpi e toru marama noa iho te pakeke, koia nā te āhuatanga o ngā pēpi kei roto nei. | Hema Temara | Te Karere | Dec 4, 2014 |
| 128 | Me kore e riro ko ia hei rangatira mō ngā iwi me ngā pā hoki o Kiharoa. | Raureti Te Huia | Mobile Unit | 1947 |
| 129 | He atamira tēnei hei whakakite atu i te reo Māori ka tahi, ngā waiata Māori ka rua, te kaha o te iwi Māori ki te waiata ka toru, i te moko konohi ka whā. | Rob Ruha | Te Karere | Dec 10, 2014 |
| 130 | Kei te hiahia kē rātou ki te hoki ki te kāinga ki te āta titiro ki ngā kōrero i puta mai i tērā wiki. | Derek Fox | Te Karere | Dec 15, 2014 |
| 131 | Me pēhea te whakatika te whakarite hōtaka wātaka o te teihana mō ngā tāngata katoa ahakoa ko wai. | Sheree Waitoa | Te Karere | Dec 2, 2014 |
| 132 | Kātahi te tangata rā ka kī atu ki ana pononga, mauria au wahangia ki Karamaina. | Te Kapa Pōtae | Mobile Unit | 1947 |
| 133 | Nā, waiho mai tērā wā taenga mai ki tēnei wā i nāianei kua waiho taua kupu hei aumihi i ngā matenga tūpāpaku. | Toki Watene | Mobile Unit | 1947 |
| 134 | Ko tēnei tangata, ko Ureia, tēnei taniwha ko Ureia, arā, ngangara, he tangata kaha ki te kai tangata. | Hoani Te Huia | Mobile Unit | 1947 |
| 135 | Ka kite atu rā te whakatinanatanga o tētahi wawata roa nei e haere ana i waenganui i te whanau. | Colin Bennett | Te Karere | Dec 1, 2014 |
| 136 | Āe rā, ka hoea atu rā te waka ka tangohia e Paikea te te punga o te waka. | Te Kapa Pōtae | Mobile Unit | 1947 |
| 137 | Kei te pito rāwhiti rawa o te o te tapa o te o te moana o Uruwhero, arā, o te wāhi e kīa nei ko te Repo o Kāwa. | Raureti Te Huia | Mobile Unit | 1947 |
| 138 | He taruhae nō te ngākau o Maru-hou-aka, rangatira o Ngāti Kauwhata, koia te pūtake i kōhurutia ai e ia a Korou-kore. | Raureti Te Huia | Mobile Unit | 1947 |
| 139 | Me nama rātou ki te whakakōrero i ō rātou utu i roto i te reo Māori nā te mea he reo mana tērā ki roto i ngā ture o Aotearoa. | Pou Temara | Te Karere | Dec 9, 2014 |
| 140 | Nā, ka nohohia a Harataunga i tēnā takiwā, ka puta te kupu a Wikitōria Anipiki. | Te Kapa Pōtae | Mobile Unit | 1947 |
| 141 | Te pā tuatahi kei te taha hauāuru ko Whiti-te-marama, te pā tuarua ko Tokanui kei waenganui, te pā tuatoru ko Pukerimu kei te taha rāwhiti. | Raureti Te Huia | Mobile Unit | 1947 |
| 142 | Nā te rongonga atu a Rongorongo ki te mea, ki te mea rā ka hoki mai ki roto ki te whare. | Māui Onekura | Mobile Unit | 1946 |
| 143 | Nā, kātahi ka tapahia e Kupete taura o te waka e Kupe te taura, te taura o te waka. | Māui Onekura | Mobile Unit | 1946 |
| 144 | He āhua uaua pea nā te mea kua kōrero mai ngā matakite Māori e haere mai ana te taniwha nei nei kānapanapa mai wāna niho koura hiriwa. | Haami Piripi | Te Karere | Dec 9, 2014 |
| 145 | Ko Tautoru ko Uruao he kotahi tonu ēnei whetū e rua e kī ana hoki tōna kōrero ka eke a Uruao ki ngā pae o Tautoru kia pupū te puna tangata me te puna kai. | Raureti Te Huia | Mobile Unit and whakapapa | 1947 |
| 146 | Ehara ko Ngāpuhi te raru i roto i te kaupapa nei, engari ko te hāunga mai o te Karauna ka rau a Ngāpuhi, nē. | Erima Henare | Te Karere | Nov 30, 2014 |
| 147 | Kātahi ka haere te kāpene, ka haere ki taha kātahi ka whakaeke a te hoari ki runga i te kakī o te koroua nei. | Te Kapa Pōtae | Mobile Unit | 1947 |
| 148 | Nā, ki taku rongo ko te take i karangatia ai taua moana ko Mānukau, ko te mānutanga anō o te waka nei o Tainui ki runga i tērā moana. | Toki Watene | Mobile Unit | 1947 |
| 149 | Te roa o te tirohanga o tētahi ā ka mutu te haerenga poka noa a ētahi ki te ata titiro ki ngā whānau. | Te Ururoa Flavell | Te Karere | Dec 2, 2014 |
| 150 | E whā ngā whare wānanga kei runga i te motu nei kei Pātea ko Matangirei ko Turi tōna tohunga kei Kāwhia ko Ahurei ko Rakataura tōna tohunga. | Raureti Te Huia | Mobile Unit | 1947 |
| 151 | E kawea mai ngā kōiwi nei me ngā upoko nei i ngā wā i te wā i te pouritanga i te rau tau e rua rau ka mahue ki muri, e tika ana kia riro mai tēnei whakatipuranga e whakatika ngā hē o te rua rau tau ki muri. | Pou Temara | Te Karere | Dec 1, 2014 |
| 152 | Ko ō rātou whānau e kōrero mai ana e kī ana kua kite au i tēnei āhuatanga kua rerekē ināianei kua āta whakarongo ia kua āta whakaaro ia ki ngā mahi tiaki anō i a mātou. | Hoturoa Kerr | Te Karere | Dec 5, 2014 |
| 153 | Nā, rokohanga atu e ia ko Kurateau, ko Kurateau e heke ana, mai ana ki waho. | Māui Onekura | Mobile Unit | 1946 |
| 154 | Nō reira, he maha ngā mea, ngā āhuatanga, i pā ki runga ki tēnei wāhi, ki runga ki tēnei whenua, ngā āhuatanga o ngā tūpuna. | Hoani Te Huia | Mobile Unit | 1947 |
| 155 | Te iritanga tēneki i te ingoa o te kīngi me te atuatanga ki runga ki a Tāwhiao. | Toroa Ngatau | Mobile Unit | 1946 |
| 156 | Nā, i taua wā e noho nei i te tangata nei a Kēneti, i Harataunga. | Te Kapa Pōtae | Mobile Unit | 1947 |
| 157 | Kātahi ka titiro atu ngā kāpene me ngā tianara ki te koroua nei e kai mai ana i te manawa o te Pākehā. | Te Kapa Pōtae | Mobile Unit | 1947 |
| 158 | Ahatia ki roto i tōku ake hapū kei reira anō ngā mea horekau e tautoko ana koia anō tēnā tō tātou āhua. | Mere Mangu | Te Karere | Nov 30, 2014 |
| 159 | He taonga mō ngā tamariki he taonga mō wā rātou uri kia tiakina rātou i te taiao kia tiakina rātou i tō tātou nei ngahere te Warawara nō reira ko te whakataukī tiakina te taiao tiakina te iwi. | Mina Pomare-Peita | Te Karere | Dec 1, 2014 |
| 160 | I taraingia e te Pākehā, ki te kiri te awa o Teawaiki, ka tae ki te wāhi he tapu kei reira, kāore e mahi he teriki, te tanuku haere tonu mai te whenua. | Hoani Te Huia | Mobile Unit | 1947 |
| 161 | He uaua nā te mea he rerekē te tirohanga o ngā mātauranga kei roto i te kura i raro i te Kawanatanga nē. | Diedre Wijohn | Te Karere | Dec 1, 2014 |
| 162 | He mōhiotanga mō tātou, e te iwi, te tino manawanui o te iwi i ērā wā ki te hāpai taonga o tō tātou ariki. | Toroa Ngatau | Mobile Unit | 1946 |
| 163 | Nā, i tētahi wā kātahi ka whawhai a Hotumō te tāhae kūmara i tētahi o wōku marae i reira. | Toki Watene | Mobile Unit | 1947 |
| 164 | Haere mai ki te tepu, kōrerohia ngā whakaaro kia rongohia ai mā tātou e whakatikatika, horekau kē pēnā ana. | Nora Rameka | Te Karere | Dec 4, 2014 |
| 165 | Ka mutu anō tōna parau i te whenua, he kō - ki te kāheru rākau a te Māori. | Raureti Te Huia | Mobile Unit | 1947 |
| 166 | Kia kore e tautohetohe whakaheahea noa iho i ngā mokopuna i ngā tuarua nē hā. | Herita Toko | Te Karere | Dec 11, 2014 |
| 167 | He tika, tētahi iwi tino nui te mana kei roto o tēnei o Ruawehea ko Ngāti Hako. | Hoani Te Huia | Mobile Unit | 1947 |
| 168 | Koinā ngā wā hapū e haere neki, i runga i te karangatia neki, i tēnei whenua, i Ruawehea. | Hoani Te Huia | Mobile Unit | 1947 |
| 169 | I te akiaki te karauna i a Tūhoronuku ahakoa te kore o te kotahitanga e pai ki aua kaupapa, i te mutunga he whaiwhai te mea i haere. | Pita Tipene | Te Karere | Nov 30, 2014 |
| 170 | Te whitinga atu ki tērā wāhi ka huri mai a Paikea ka tapaina te ingoa o tēnā whenua mai i tērā rā ki tēnei rā ko Tauranga. | Te Kapa Pōtae | Mobile Unit | 1947 |
| 171 | E kore tēnei kaupapa te whakatutuki i te kereme a Ngāpuhi e oti kia whakahoungia katoangia ngā mea katoa. | Tukoroirangi Morgan | Te Karere | Dec 2, 2014 |
| 172 | Nō tēnei wā hoki i tukuna ai e Te Wherowhero tēnā whenua, a Moeawhā, ki a Te Mōkena hei whenua kura hei ako i ngā tamariki ki te ngaki whenua. | Raureti Te Huia | Mobile Unit | 1947 |
| 173 | Nā, mai i taua rā tae mai ki tēnei rā ko te ingoa o tēnei motu ko Ahuahu. | Te Kapa Pōtae | Mobile Unit | 1947 |
| 174 | Nā tēnei, tēnei anō te take i haere mai ai a Turi ki tēnei motu. | Māui Onekura | Mobile Unit | 1946 |
| 175 | Ā, ki te ako hoki i ngā waiata i ngā haka i ngā pātere i ngā ruriruri i ngā oriori kei runga tonu i te āhua o te o te wāhine te oti ai ēnei hanga. | Raureti Te Huia | Mobile Unit | 1947 |
| 176 | E awangawanga ahau i te wā nei engari e hari koa ana hoki ahau nā te mea he mea nui tēnei ki a au. | Moana Te Hei | Te Karere | Dec 3, 2014 |
| 177 | Ko te taniwha ko Tuputetaiheke he tangata haere tēneki, he tangata tūtūtohu hoki i ngā pakanga ka whakaekengia ana a Ngāti Tamaterā. | Hoani Te Huia | Mobile Unit | 1947 |
| 178 | Nā rātou tonu i tuku tēnei kaupapa ki te katoa i te tīmatatanga o tēnei tau. | Derek Fox | Te Karere | Dec 15, 2014 |
| 179 | Hei whakarongo mai mā koutou mō ētahi wāhi tautau kōrero i waenganui i tēnei karangatanga e karangatia neki, ko Ruawehea | Hoani Te Huia | Mobile Unit | 1947 |
| 180 | Ka noho a Te Kōhi ki Mangatawhiri ko ngā pao tēnei, arā, ko ngā haka o ērā rangi nā te taha Kīngitanga tēnei haka. | Raureti Te Huia | Mobile Unit | 1947 |
| 181 | Mā ngā tauira i roto i ngā kōrero i ngā mahi a ō tātou tūpuna ka puta mai te ngakau mahaki te ngakau whakaora tangata. | Hoturoa Kerr | Te Karere | Dec 5, 2014 |
| 182 | Engari i tēnei rā i te taenga o Paikea ki reira e kī āna tai. | Te Kapa Pōtae | Mobile Unit | 1947 |
| 183 | Ko te rori hoki tēnei o Mangatawhiri me Pōkeno kei reira a Te Tōhi e noho mai ana kei te kati taha kīngitanga. | Raureti Te Huia | Mobile Unit | 1947 |
| 184 | Nā, te matenga anō o taua tamaiti, pokaina ana e Turi te manawa, haria ana ki te amo a Hotukura ki te ariki, ki a Uenuku. | Māui Onekura | Mobile Unit | 1946 |
| 185 | Nā, e pari mai ana te tai ka kī atu ngā wāhine me noho rātou kia timu te tai kia whakawhiti ai. | Te Kapa Pōtae | Mobile Unit | 1947 |
| 186 | Take i tae mai ai a Ngāti Porou ki tēnei whenua ki Hauraki, arā, ki Harataunga. | Te Kapa Pōtae | Mobile Unit | 1947 |
| 187 | Ko Waiharakeke te awa i tupu ai a Aotea, nā Toto i turaki, ka hinga ki te whenua. | Māui Onekura | Mobile Unit | 1946 |
| 188 | Nā, i taua takiwā, kua nui ngā tāngata kua mōhio ki te kaumātua nei e whai moni ana a ia. | Te Kapa Pōtae | Mobile Unit | 1947 |
| 189 | I whakahokia ki ngā Māori kāore nei i tika ki aua whenua, ā ko ētehi hoki o ēnei whenua kei te takoto noa kāore kau ana he tangata he tangata o runga | Raureti Te Huia | Mobile Unit | 1947 |
| 190 | Me kī ko te mahi tika i te mahi kia kore te waka e tāhuri kia kore e wīwī e wāwā te haere. | Piripi Houia | Te Karere | Dec 11, 2014 |
| 191 | Ā, ka riro nā te tūāraki i whakahuri maiki roto ki te moana e kīa nei e ō tātou tūpuna i tēnei wā ko Tīkapa. | Toki Watene | Mobile Unit | 1947 |
| 192 | Ki te kore ki te 50 paihēneti e eke he tūru etahi tūru kia hiki te mana o te reo Māori ki roto i tēnei tū āhuatanga. | Marama Fox | Te Karere | Dec 3, 2014 |
| 193 | Kei Pōneke e noho ana Te Raipere he Māori ōna kōrero kei reira e noho ana, me ngā whakahaere o te waka nei, o Turi. | Māui Onekura | Mobile Unit | 1946 |
| 194 | Heoi anō kia whiwhia ai e mātou he wāhanga i roto i ngā āhuatanga o te mahi nei kia tutuki noa ā mātou nei mahi kaitiaki i runga i te moana i runga i te whenua anō hoki, te papa moana koia nā āhuatanga katoa. | Haami Piripi | Te Karere | Dec 9, 2014 |
| 195 | Ko ētehi o aua whenua he mea hoko nā ngā tāngata kē, ehara nei i a rātou te whenua. | Raureti Te Huia | Mobile Unit | 1947 |
| 196 | Kātahi te wheke, te wheke rā, ka maranga ki runga i te waka o Kupe. | Māui Onekura | Mobile Unit | 1946 |
| 197 | Nōku te whiwhi ki te tipu mai i te mātotorutanga o te reo Māori me ōna tikanga. | Apirana Pewhairangi | Te Karere | Nov 30, 2014 |
| 198 | Āe wāku mokopuna, ā ko tāku i tēnei wā kia kua e heke ngā pakanga ngā kōhimu ngā āhuatanga pai kia kaua e heke ki wāku tamariki. | Jennifer Matamua | Te Karere | Dec 17, 2014 |
| 199 | Kua nukuhia ētahi o te whānau, engari i ngā tau e rua kua pahure nei kei te tirotiro mai ngā kōhanga o te takiwā nei. | Katene Paenga | Te Karere | Dec 12, 2014 |
| 200 | Koia rā tonu te kaupapa, ko ngā mokopuna ko te reo me te tapu o te reo | Huirangi Waikerepuru | Te Karere | Dec 11, 2014 |

**References**

Anderson, A. 1989 *Prodigious birds - Moas and moa-hunting in prehistoric New Zealand*. Cambridge, Cambridge University Press.

Bagnall, A. G., G. C. Petersen, and I. St. George. 2012 *William Colenso: Printer, missionary, botanist, explorer, politician: His life and journeys*. Dunedin, Otago University Press.

Berkes, F. 2008. *Sacred ecology*. Vol. 2nd. New York: Routledge.

Best, E. 1922 *The astronomical knowledge of the Maori, genuine and empirical: Including data concerning their systems of astrogeny, astrolatry, and natural astrology, with notes on certain other natural phenomena*. *Dominion Museum monograph no. 3*. Wellington, Government Printer.

Best, E. 1923 Obituary - Stephenson Percy Smith. 1840-1922. *Transcations and Proceeding of the New Zealand Institute* **54**, xiii-xiv.

Best, E. 1924a *The Maori as he was: A brief account of Maori life as it was in pre-European days*. Wellington, Government Printer.

Best, E. 1924b *Maori religion and mythology: Being an account of the cosmogony, anthropogeny, religious beliefs and rites, magic and folk lore of the Maori folk of New Zealand*. *Bulletin / New Zealand Dominion Museum*. Wellington, Government Printer.

Best, E. 1942 *Forest lore of the Maori: With methods of snaring, trapping, and preserving birds and rats, uses of berries, roots, fern-root, and forest products, with mythological notes on origins, karakia used etc*. *Dominion Museum bulletin no. 14*. Wellington, Polynesian Society in collaboration with Dominion Museum.

Best, E. 1986 *The Maori school of learning: Its objects, methods, and ceremonial*. *Dominion Museum monograph no. 6*. Wellington, Government Printer.

Best, E. 1925. *Tuhoe, the children of the mist: A sketch of the origin, history, myths and beliefs of the Tuhoe tribe of the Maori of New Zealand, with some account of other early tribes of the Bay of Plenty district*. *Memoirs of the Polynesian Society v.6*. Wellington, Board of Maori Ethnological Research for the Author and on behalf of the Polynesian Society.

Best, E., and the New Zealand Institute. 1907 *Maori forest lore: Being some account of native lore and woodcraft, as also of many myths, rites, customs and superstitions connected with the flora and fauna of the Tuhoe and Ure-wera district. Part 1*. Auckland, Auckland Institute.

Best, E., and New Zealand Dept. of Lands and Survey. 1897 *Waikare-moana, the sea of the rippling waters: The lake, the land, the legends: with a tramp through Tuhoe land*. Wellington, Government Printer.

Biggs, B. 1952 The translation and publishing of Maori material in the Auckland Public Library. *The Journal of the Polynesian Society* **61**, 176-191.

Buick, T. L. 1937 *The moa-hunters of New Zealand: Sportsmen of the Stone Age*. New Plymouth, Thomas Avery.

Buller, W. L. 1888 *A history of the birds of New Zealand*. London, The Author.

Byrnes, G. M. 2014 Smith, Stephenson Percy – Biography’, from the Dictionary of New Zealand Biography. Te Ara - the Encyclopedia of New Zealand. URL: http://www.TeAra.govt.nz/en/biographies/2s33/smith-stephenson-percy. In *Smith, Stephenson Percy – Biography’, from the Dictionary of New Zealand Biography. Te Ara - the Encyclopedia of New Zealand. URL:* http://www.TeAra.govt.nz/en/biographies/2s33/smith-stephenson-percy.

Colenso, W. 1844 *Excursion in the Northern Island of New Zealand in the summer of 1841–42*. Launceston, Tasmania, Land.

Colenso, W. 1868 On the Maori races of New Zealand. *The New Zealand Institute* **1**, 339-423.

Colenso, W. 1878 Contributions to a better knowledge of the Maori race. Part I: Legends, myths and fables; The Iideality of the ancient New-Zealander. *The New Zealand Institute* **11**, 77-106.

Colenso, W. 1879 Contributions towards a better knowledge of the Maori race. Part II: On the ideality of the ancient New-Zealander; Proverbs and proverbial sayings. *The New Zealand Institute* **12**, 108-147.

Colenso, W. 1884 *An account of visits to and crossings over the Ruahine mountain range, Hawke's Bay, New Zealand: and of the natural history of that region, 1845-1847*. Napier, New Zealand, Daily Telegraph.

Cowan, J. 1935 Famous New Zealanders: No. 31: Mr. S. Percy Smith: Pioneer surveyor, explorer, ethnologist and historian. *The New Zealand Railways Magazine* **10**, 21-24; 46.

Crombie, W. 1985a *Discourse and language learning: A relational approach to syllabus design*. Oxford, Oxford University Press.

Crombie, W. 1985b *Process and relational discourse and language learning.* Oxford, Oxford University Press.

Crombie, W. 1987 *Free verse and prose style*. London; New York; Sydney, Croom Helm.

Curnow, J. 1985 Wiremu Maihi Te Rangikaheke: His life and work. *The Journal of the Polynesian Society* **94**, 97-148.

Curnow, J. 2002 A brief history of Maori-language newspapers. In *Rere Atu, Taku Manu!: Discovering history, language & politics in the Maori-language newspapers, 1842-1933*, edited by J. Curnow, N. Hopa, and J. McRae, pp. 17-41. Auckland, New Zealand, Auckland University Press.

Curnow, J., N. Hopa, and J. McRae. 2002 *Rere Atu, Taku Manu!: Discovering history, language & politics in the Maori-language newspapers, 1842-1933*. Auckland, New Zealand, Auckland University Press.

Dunning, J. B. 2007 *CRC Handbook of Avian Body Masses*. Hoboken, CRC Press.

Firth, R. W. 1926. Proverbs in native life, with special reference to those of the Maori, Parts I and 2. *Folklore* **37**, 134-153; 245-270.

Foster, B., J. 2011 White, John – Biography’, from an Encyclopedia of New Zealand edited by A. H. McLintock, orignally published in 1966. URL: http://www.TeAra.govt.nz/en/1966/white-john. In *White, John – Biography’, from an Encyclopedia of New Zealand edited by A. H. McLintock, orignally published in 1966. URL:* http://www.TeAra.govt.nz/en/1966/white-john.

Grey, G. 1853 *Ko nga moteatea, me nga hakirara o nga Maori: He mea kohikohi mai*. Wellington, Printed by Robert Stokes.

Grey, G. 1857 *Ko nga whakapepeha me nga whakaahuareka a nga tipuna o Aotea-roa: Proverbial and popular sayings of the ancestors of the New Zealand race*. Cape Town, Saul Solomon.

Grey, G. 1870 Letter from, containing remarks on a letter from Dr. Haast. *Proceedings Zoological Society of London*, 116-117.

Grey, G. 1971 *Nga mahi a nga tupuna*, 4th edition. Wellington, A.H. & A.W. Reed.

Griffith, P., R. Harvey, and K. Maslen. eds. 1997 *Book & print in New Zealand: A guide to print culture*. Wellington, New Zealand: Victoria University Press.

Grove, N. 1981. *Ngā pēpeha a ngā tīpuna*. Wellington, Department of Maori Studies, Victoria University of Wellington.

Hutton, F. W., and M. Coughtrey. 1875 Notice of the Earnscleugh Cave. *Transcations and Proceeding of the New Zealand Institute* **7**, 138-144.

McRae, J. 2014 Māori newspapers and magazines – ngā niupepa me ngā moheni - Role of Māori newspapers. Te Ara - the Encyclopedia of New Zealand. URL: http://www.TeAra.govt.nz/en/maori-newspapers-and-magazines-nga-niupepa-me-nga-moheni/page-1. In *Māori newspapers and magazines – ngā niupepa me ngā moheni - Role of Māori newspapers. Te Ara - the Encyclopedia of New Zealand. URL:* http://www.TeAra.govt.nz/en/maori-newspapers-and-magazines-nga-niupepa-me-nga-moheni/page-1.

Mead, S. M., and N. Grove. 1989 *Ngā pēpeha a ngā tīpuna: Te wahanga 1*. Wellington, Department of Maori Studies, Victoria University of Wellington.

Mead, S. M., and N. Grove. 1991 *Ngā pēpeha a ngā tīpuna: Te wahanga 2*. Wellington, Department of Maori Studies, Victoria University of Wellington.

Mead, S. M., and N. Grove. 1994 *Ngā pēpeha a ngā tīpuna: Te wahanga 3*. Wellington, Department of Maori Studies, Victoria University of Wellington.

Mead, S. M., and N. Grove. 2001. *Ngā pēpeha a ngā tīpuna: The sayings of the ancestors*. Wellington, Victoria University Press.

O’Leary, J. 2008 John White, 1826 – 1891 *Kōtare* **2**, 48-54.

Owen, R. 1839 On the bone of an unknown Struthious bird from New Zealand *PZSL* **VII**.

Owen, R. 1879 *Memoirs on the extinct wingless birds of New Zealand, with an appendix on those of England, Australia, Newfoundland, Mauritius, and Rodriguez*. London, John van Voorst.

Perry, G. L. W., A. B. Wheeler, J. R. Wood, and J. M. Wilmshurst. 2014 A high-precision chronology for the rapid extinction of New Zealand moa (Aves, Dinornithiformes). *Quaternary Science Reviews* **105**, 126-135. 10.1016/j.quascirev.2014.09.025.

Polack, J. 1838 *New Zealand, being a narrative of travels and adventures (2 vols.)*. London, Richard Betley.

Porter, F. 2014 Williams, William – Biography’, from the Dictionary of New Zealand Biography. Te Ara - the Encyclopedia of New Zealand. URL: URL: http://www.TeAra.govt.nz/en/biographies/1w26/williams-william. In *Williams, William – Biography’, from the Dictionary of New Zealand Biography. Te Ara - the Encyclopedia of New Zealand. URL:URL:* http://www.TeAra.govt.nz/en/biographies/1w26/williams-william.

Reilly, M. P. J. 2014 White, John – Biography’, from the Dictionary of New Zealand Biography. Te Ara - the Encyclopedia of New Zealand. URL: http://www.TeAra.govt.nz/en/biographies/1w18/white-john. In *White, John – Biography’, from the Dictionary of New Zealand Biography. Te Ara - the Encyclopedia of New Zealand. URL:* http://www.TeAra.govt.nz/en/biographies/1w18/white-john.

Roa, T. 2016 An exploration of the role of semantic relations in the theory and practice of translation (with special reference to English/Māori and Māori/English translation). Doctor of Philosophy (PhD), Faculty of Māori and Indigenous Studies, University of Waikato, Hamilton, New Zealand.

Simmons, D. 1966 The sources of Sir George Grey's Nga mahi a nga tupuna. *The Journal of the Polynesian Society* **75**, 177-188.

Sinclair, K. 1983 The origins of the species. In *The Summer Book 2*, edited by B. Williams, R. Parsons, and L. MIssens, pp. 27-32. Wellington, The Port Nicholson Press.

Sissons, J. 2010 Best, Elsdon – Biography’, from the Dictionary of New Zealand Biography. In *Best, Elsdon – Biography’, from the Dictionary of New Zealand Biography. Te Ara - the Encyclopedia of New Zealand. URL:* http://www.TeAra.govt.nz/en/biographies/2b20/1.

Smith, S. P. 1898 *Hawaiki: The whence of the Maori; with a sketch of Polynesian history*. Wellington, Whitcombe & Tombs.

Smith, S. P. 1904a *Hawaiki: The original home of the Maori; with a sketch of Polynesian history*. Wellington, Whitcombe & Tombs.

Smith, S. P. 1904b *Wars of the northern against the southern tribes of New Zealand in the nineteenth century*, 2nd edition. Wellington, Whitcombe & Tombs.

Smith, S. P. 1910 *History and traditions of the Maoris of the West Coast, North Island of New Zealand, prior to 1840*. New Plymouth, Printed for the Society by T. Avery.

Smith, S. P., H. T. Whatahoro, Te Matorohanga, and N. Pohuhu. 1913 *The Lore of the Whare-waananga, or, Teachings of the Maori college on religion, cosmogony and history: Vol 1: Te Kauwae-runga, or 'things celestial'*. *Memoirs of the Polynesian Society*. New Plymouth, Printed for the Society by T. Avery.

Smith, S. P., H. T. Whatahoro, Te Matorohanga, and N. Pohuhu. 1915 *The Lore of the Whare-waananga, or, Teachings of the Maori college on religion, cosmogony and history: Vol 2: Te Kauwae-raro, or 'things terrestrial'*. *Memoirs of the Polynesian Society*. New Plymouth, Printed for the Society by T. Avery.

Tennyson, A. 2006 *Extinct birds of New Zealand*. Wellington, Te Papa Press.

Tregear, E. 1922 Obituary - The late Stephenson Percy Smith, president and founder of the Polynesian Society and editor of its journal. *Journal of the Polynesian Society* **31**, 67-75.

Wehi, P. M. 2009 Indigenous Ancestral Sayings Contribute to Modern Conservation Partnerships: Examples Using Phormium Tenax. *Ecological Applications* **19**, 267-275. 10.1890/07-1693.1.

Whaanga, H. 2007 Inter-propositional relations: An investigation of authentic Māori texts. *Journal of Maori and Pacific Development* **8**, 54-82.

Whaanga, J. P. 2006 Case roles/relations and discourse relations: A Māori language-based perspective. Doctor of Philosophy (PhD), School of Māori and Pacific Development, University of Waikato, Hamilton, New Zealand.

White, J. 1887-1890 *The ancient history of the Maori, his mythology and traditions (Vols 1-6)*. Wellington, Government Print.

Wood, J. R. 2013 Extinction - New Zealand, 500 years ago. In *Grzimek’s Animal Life Encyclopedia*, edited by N. MacLeod, J. D. Archibald, and P. Levin, pp. 595-604. Chicago, Booklist Publications.
